# Cardiovascular Mortality Gap Between the United States and Other High Life Expectancy Countries in 2000–2016

**DOI:** 10.1093/geronb/gbac032

**Published:** 2022-02-23

**Authors:** Enrique Acosta, Neil Mehta, Mikko Myrskylä, Marcus Ebeling

**Affiliations:** Max Planck Institute for Demographic Research, Rostock, Germany; Department of Preventive Medicine and Population Health, The University of Texas Medical Branch, Galveston, Texas, USA; Max Planck Institute for Demographic Research, Rostock, Germany; Center for Social Data Science, University of Helsinki, Helsinki,Finland; Max Planck Institute for Demographic Research, Rostock, Germany; Unit of Epidemiology, Institute of Environmental Medicine, Karolinska Institutet, Stockholm, Sweden

**Keywords:** Cardiovascular disease, Decomposition, Mortality, Obesity, Trend analysis

## Abstract

**Objectives:**

Reductions in U.S. cardiovascular disease (CVD) mortality have stagnated. While other high life expectancy countries (HLCs) have also recently experienced a stall, the stagnation in CVD mortality in the United States appeared earlier and has been more pronounced. The reasons for the stall are unknown. We analyze cross-national variations in mortality trends to quantify the U.S. exceptionality and provide insight into its underlying causes.

**Methods:**

Data are from the World Health Organization (2000–2016). We quantified differences in levels and trends of CVD mortality between the United States and 17 other HLCs. We decomposed differences to identify the individual contributions of major CVD subclassifications (ischemic heart disease [IHD], stroke, other heart diseases). To identify potential behavioral explanations, we compared trends in CVD mortality with trends in other causes of death related to obesity, smoking, alcohol, and drugs.

**Results:**

Our study has four central findings: (a) U.S. CVD mortality is consistently higher than the average of other HLCs; (b) the U.S.–HLC gap declined until around 2008 and increased thereafter; (c) the shift from convergence to divergence was mainly driven by slowing IHD and stroke mortality reductions and increasing mortality from other CVD causes; (d) among the potential risk factors, only obesity- and alcohol-related mortality showed age-specific temporal changes that are similar to those observed for cardiovascular mortality.

**Discussion:**

The exceptional changes in U.S. CVD mortality are driven by a distinct pattern of slowing reductions in IHD and stroke mortality and deteriorating mortality from other CVD causes. Obesity and alcohol abuse appear to be interrelated factors.

Since around 2010, U.S. life expectancy improvements have stalled, and during 2014–2017, the United States experienced three consecutive year-over-year declines. Such a trend reversal has not been observed in a century. The reasons for this deterioration are not yet completely understood, and the drivers of this process are likely multidimensional (National Academies of Sciences, Engineering, and Medicine [NASEM] 2013, 2021). The so-called “deaths of despair”—deaths from suicides and drug and alcohol abuse—are often seen as central drivers of the recent trend (Barbieri, 2019; Case & Deaton, 2017, 2020; Masters et al., 2018). Drug overdose mortality, for instance, has increased exponentially since 2000 (Ho, 2019, 2020; Jalal et al., 2018).

Recent research also points to cardiovascular disease (CVD) mortality as the main factor in recent U.S. mortality dynamics. CVD-related deaths make up almost one third of all deaths in the United States. Thus, changes in CVD mortality have an enormous effect on overall life expectancy trends. Sharp reductions in CVD mortality have been the main driver of the overall mortality decline in the United States and other high life expectancy countries (HLCs) since the 1970s, a period that has also been called the “cardiovascular revolution” (Grigoriev et al., 2014; Vallin & Meslé, 2004).

Previous research, however, has established that most HLCs have recently faced a deceleration in CVD mortality declines (Lopez & Adair, 2019). Figure 1 depicts the age-standardized death rates (ASDRs) from CVD in the United States and 17 other HLCs. Although CVD mortality reductions have decelerated in most countries, the United States stands out from this pattern, with the stall appearing earlier, starting around 2008; and the magnitude of the stall being more pronounced. Several authors have documented that CVD mortality has decreased very little in the United States (Lopez & Adair, 2019; Shah et al., 2019, 2020) since around 2010 and have argued that it might have contributed substantially to the increase in all-cause mortality (Barbieri, 2019; Woolf & Schoomaker, 2019). A recent analysis conducted by Mehta et al. (2020) concluded that the deceleration in CVD mortality reductions was responsible for the stagnation in U.S. life expectancy.

**Figure 1.**
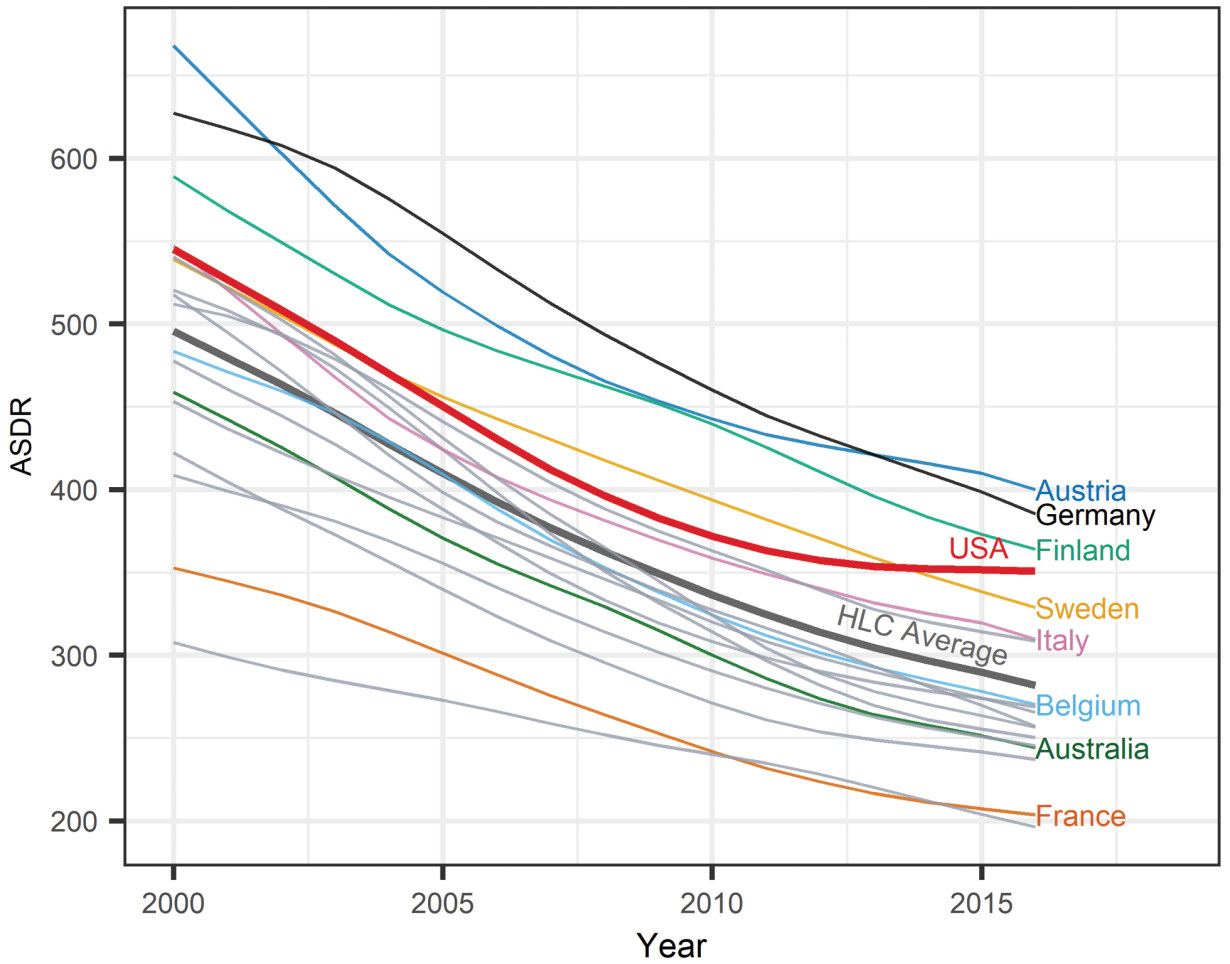
Age-standardized death rates (ASDRs) from cardiovascular disease across high life expectancy countries (HLCs). The U.S. population in 2016 is used as the reference. The countries included in this analysis are the United States, Australia, Austria, Belgium, Canada, Denmark, Finland, France, Germany, Italy, Japan, the Netherlands, New Zealand, Norway, Spain, Sweden, Switzerland, and the United Kingdom.

The patterns of CVD mortality observed in other countries, such as Belgium, France, and Japan, indicate that decreases in CVD-related mortality in the United States have not reached their limit. These countries have lower levels of CVD mortality than the United States and have continued to experience robust CVD mortality declines in the absence of medical innovation.

Several hypotheses have been suggested to explain the stalling of the CVD slowdown in the United States. *First, the recent stagnation in CVD-related mortality in the United States may be attributable to the ongoing obesity epidemic* (Barbieri, 2019; Mehta et al., 2020; Sidney et al., 2016). Thus, the obesity epidemic might have reached a point where its detrimental impact has become visible in population-level mortality trends for related diseases, such as CVD. *Second, the drug abuse epidemic may have exacerbated CVD-related mortality.* Both the synchronicity of these developments and the mounting evidence that substance abuse is a risk factor for CVD suggest a potential association (Day et al., 2019; Glei & Preston, 2020; Khodneva et al., 2016; Spadotto et al., 2013). *Third*, *the observed CVD stagnation in the United States may be partially driven by recent increases in alcohol abuse.* A substantial surge in alcohol-related mortality in the United States since 2000 has been observed (Spillane et al., 2020; Vierboom, 2020; White et al., 2020). Although moderate alcohol consumption may be protective against CVD mortality (Ronksley et al., 2011), alcohol abuse has been associated with increased CVD deaths (Bell et al., 2017; Di Cesare et al., 2013; Klatsky, 2015; Stokes & Preston, 2017). *Fourth, shrinking opportunities for mortality reductions due to the smoking decline may be causing the stalling of CVD mortality* (Lopez & Adair, 2019; Mehta et al., 2020). The decline in smoking has been a key factor in past U.S. mortality reductions, particularly in CVD mortality (Grigoriev et al., 2014). However, as smoking in the United States has decreased steadily in recent decades, most opportunities for smoking-related mortality reductions may have already been realized. *Finally*, while several further explanations have been proposed, the likelihood that there is a *systemic cause affecting mortality from several causes, including CVD*, has been repeatedly emphasized (NASEM, 2021). A systemic cause might result from the interplay of psychological and socioeconomic distress factors, such as the widening inequalities in the United States, which could be a trigger for “deaths of despair,” obesity, and the stagnation in CVD-related mortality, among other effects (Mehta et al., 2020).

In this study, we leverage cross-national variation in CVD mortality trends and trends in causes of death that share similar risk factors (obesity, smoking, alcohol, and drugs). We ask three main questions. First, how do the temporal dynamics of U.S. CVD mortality compare to those in other HLCs? Second, what are the relative contributions of major CVD subclassifications (ischemic heart disease, stroke, and other heart diseases) to the differences in CVD mortality trends between the United States and the other HLCs? Third, how do the changes in CVD mortality in the United States and other HLCs compare to changes in mortality that are related to known behavioral risk factors, including obesity, smoking, alcohol, and drug abuse?

## Method

### Data

We analyze the dynamics of the differences in CVD mortality between the United States and 17 other HLCs using cause-of-death data from the World Health Organization (WHO) mortality database (WHO, 2020). The countries are selected based on three criteria: (a) their life expectancy was at least that of the United States for both sexes combined in 2000–2005, according to the World Population Prospects (WPP) estimates (WPP, 2019); (b) they are considered *developed economies* according to the UN country classifications (DESA-UN, 2020); and (c) they have at least 14 years of age-, sex-, and cause-specific death data between 2000 and 2016 in the WHO database. The list of countries is provided in the caption to Figure 1 and Supplementary Table 1. Our analysis spans the years 2000–2016, a period for which mortality data based on the 10th Revision of the International Classification of Diseases (ICD-10) are available for most of the countries under analysis.

Causes of death are coded based on the three-digit version of the ICD-10. Annual population estimates for the computation of death rates are retrieved from the WPP database (WPP, 2019).

### Analytical Strategy

**Figure 2.**
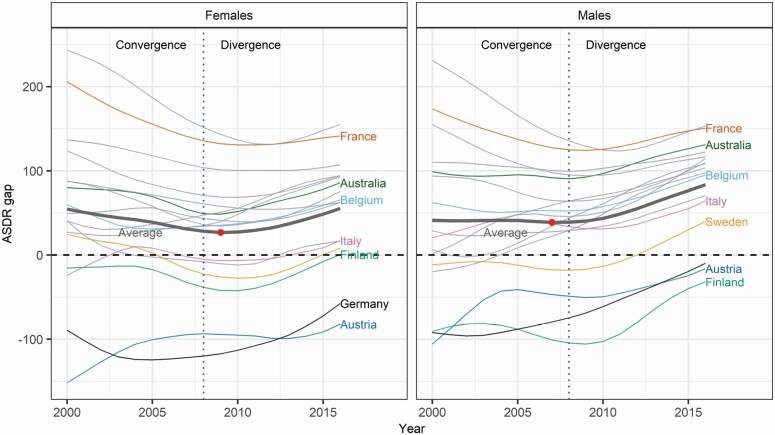
Gap in age-standardized death rates (ASDRs) from CVD between the United States and other HLCs by sex. The U.S.–HLC average gap is indicated with a thick line. The dotted vertical line indicates the year 2008, when the U.S.–HLC gap shifted from convergence to divergence. The U.S. population in 2016 is used as the reference. CVD = cardiovascular disease; HLCs = high life expectancy countries.

Based on the observation that the gap in the ASDR between the United States and other countries largely follows a U-shaped pattern (Figure 2), we decided to use the sex-specific HLC average for comparison. The average provides a parsimonious alternative to a country-by-country comparison and thus enables us to compare the United States against a general international high life expectancy trend.

#### Data preparation

The analysis is based on death rates grouped by calendar year, 5-year age groups, sex, country, and cause of death. We restrict our analysis to the 20 to 85+ age groups to allow for comparability across countries and over time. To reduce random fluctuations, death rates are smoothed independently over time for each age, sex, country, and cause of death using cubic splines. In four countries, mortality data are not available for the full 2000–2016 period. In Australia, death data are not available for 2005; while in the United Kingdom, Austria, and Italy, data are available only after 2000, 2001, and 2002, respectively (Supplementary Table 1). Age-, sex-, and cause-specific death rates for the missing years are obtained from cubic splines inter- and extrapolations. We also perform two robustness checks: (a) using the original data series, excluding the periods with missing data; and (b) excluding these four countries from the analyses. The results from these sensitivity analyses, presented in the Supplementary Figures 7–14, are entirely consistent with the findings presented here.

We analyze mortality for all CVD deaths together (all CVD, I00–I99) and separately for three major subclassifications: ischemic heart diseases (IHD, I20–I25), stroke (I61–I64), and all other CVD causes combined. Nevertheless, for completeness, we also present results that are additionally stratified by hypertensive diseases (I10–I15), heart failure (I50), other cerebrovascular diseases (I60, I65–I69), circulatory diseases (I70–I99), and other heart diseases in Supplementary Figures 3, 5, and 6.

We also analyze how changes in CVD mortality correlate with changes in mortality from four other cause-of-death groups with similar risk factors: smoking-related (lung cancer and chronic obstructive pulmonary disease), obesity-related (obesity-related cancers, diabetes, overweight, and obesity), alcohol-related (behavioral disorders due to alcohol, alcoholic liver disease, accidental poisoning by alcohol, chronic hepatitis not elsewhere classified, fibrosis and cirrhosis of the liver), and drug-related causes of death (drug overdoses and mental and behavioral disorders due to psychoactive substance use). We present a detailed list of the ICD-10 codes employed to define the causes of death in Supplementary Table 2.

#### Decomposing the CVD mortality gap

Our comparison of the United States with the average of 17 other HLCs provides the ASDRs. The ASDR for time *t* is calculated by $ASDR_{t}=\sum_{x}\;c_{St.,x}m_{x,t}$, with $c_{St.,\;x}$ being the age-specific (*x*) weights of the U.S. population in 2016, which we use as the standard population (*s_t_*), and *m_x,t_* being the age group-specific death rate at time *t*. We calculate the ASDR separately for each sex and cause of death.

The ASDR gap between the United States and the HLC average shows two distinct periods: a period of CVD mortality convergence during 2000–2008 and a period of divergence during 2008–2016, with 2008 being the mid-point between male and female years of the trend change.

We decompose the mortality difference and its change in three steps that we label *gap* (*G*), *gap trend* (*GT*), and *convergence to divergence shift* (*CDS*). With this decomposition, we aim to examine the temporal dynamics of the U.S.–HLC gap in CVD mortality and to identify the cause-specific contribution to the shift from mortality convergence to divergence.

The *gap* (*G*) in ASDR at each time point is obtained by subtracting CVD mortality in the HLC average from that in the United States,


$$G_{t}=\sum_{x}\;c_{St.,\;x}(m_{x,t}^{\text{US}}-m_{x,t}^{\text{HLC}})$$ (1)

with *m_x,t_* being the CVD death rate for the United States and the HLC average at age group *x* and time point *t*. The *gapG* is interpreted as a weighted average of the age-specific mortality differences.

In the second step, we estimate the *GT* separately for the periods with mortality convergence (2000–2008) and mortality divergence (2008–2016), labeled GT_C_ and GT_D_, respectively. This is done by subtracting the gap in the more recent year from the gap in the earlier year, separately for the two periods,


$${\text{GT}}_{C}={\text{GT}}_{2008}-{\text{GT}}_{2000}=\sum_{x}\;c_{St.,\;x}\left[\left(m_{x,2008}^{\text{US}}-m_{x,2008}^{\text{HLC}}\right)-(m_{x,2000}^{\text{US}}-m_{x,2000}^{\text{HLC}})\right]$$ (2)

$${\text{GT}}_{D}={\text{GT}}_{2016}-{\text{GT}}_{2008}=\sum_{x}\;c_{St.,\;x}\left[\left(m_{x,2016}^{\text{US}}-m_{x,2016}^{\text{HLC}}\right)-(m_{x,2008}^{\text{US}}-m_{x,2008}^{\text{HLC}})\right]$$

The *GT* is interpreted as the weighted average of the change in age-specific mortality differences between the respective periods, indicating the trend of the mortality differential. In the third step, we compute the *convergence-to-divergence shift* (*CDS*) by subtracting the *GT* during mortality convergence (GT_C_) from that during mortality divergence (GT_D_),


$$\text{CDS}={\text{GT}}_{\text{D}}-{\text{GT}}_{\text{C}}.$$ (3)

*CDS* expresses how the trend in the mortality differences changed between the periods of mortality convergence and divergence. As the three steps described in Equations 1–3 consist simply of subtractions of ASDRs or age-specific death rates, the presented decomposition can easily be extended to also include cause-specific contributions. This is done by also summing, in addition to age, over the cause-specific death rates ($m_{x,t}^{\text{CoD}}$) (i.e., ${\text{ASDR}}_{t}=\sum_{x}\;\sum_{\text{CoD}}\;c_{St.,x}m_{x,t}^{\text{CoD}}$). This additive property of the ASDR allows us to analyze the isolated age- and cause-specific contributions.

#### Linking CVD mortality change to change in mortality from other causes

In the second part of the analysis, we compare the mortality trends in IHD, stroke, and other CVD with the mortality trends of the causes of death that share similar risk factors (i.e., smoking, obesity, alcohol, and drugs). The mortality trends during the period of mortality convergence and divergence are measured by averaging annual mortality changes within each period. We perform two different types of analyses. First, we assess the correlation of age- and sex-specific average annual mortality changes during the convergence and divergence periods between each of three CVD groups and the four different risk factor–related mortality groups. We use the nonparametric Spearman’s rank correlation measure, as it does not depend on the distribution of the data. The correlation indicates to what extent different causes of death exhibit similar paces of mortality change across age, sex, and periods. Second, we compare the age profiles of the average annual mortality changes between the convergence and divergence periods in the United States and HLCs by visually exploring the age-specific mortality trend changes for each CVD cause and risk factor–related mortality group. This analysis allows us to evaluate the age-specific mortality dynamics among the CVD cause groups, and how they compare to those observed for the risk factor–related groups.

All of the data and code for reproducing results are openly available (Acosta et al., 2021).

## Results

The ASDRs depicted in Figure 1 indicate that in 2000, the United States had higher CVD mortality than most other HLCs. Only Austria, Germany, and Finland had higher levels.

Figure 2 depicts the annual gap in CVD mortality (in ASDRs) between the United States and other HLCs, hereafter called the U.S.–HLC gap. The U.S.–HLC gap displays a U-shaped pattern over time for most countries. In the early 2000s, CVD mortality was declining faster in the United States than in other countries, leading to a convergence. However, U.S. CVD mortality reductions slowed over time, leading to a divergence of U.S. CVD mortality levels from those of most other HLCs. Although CVD mortality reductions decelerated in most countries and in the HLC average, the slowdown was most noticeable in the United States. For instance, between 2000–2008 and 2008–2016, the decrease in the annual rate of mortality reduction was four times higher in the United States than in the HLC average, and 7.2 percentage points higher than that in Austria, the country with the second-highest decrease (Supplementary Table 3). Between the two periods, CVD mortality reductions accelerated only in Finland, Germany, and Japan. As mentioned earlier in the *Analytical Strategy* section, given the similar shape of the U.S.–HLC gaps over time and across countries, we chose to compare U.S. CVD mortality with that obtained by averaging the cause-specific death rates across HLCs. Based on this set of reference death rates, we identified 2009 (females) and 2007 (males) as the years with the smallest gap in the CVD ASDR. To ensure coherence between the analyses of both sexes and compare periods with identical timespans, we have chosen 2008 as the breakpoint between CVD mortality convergence and divergence. Supplementary Figure 1 presents the U.S.–HLC gap for IHD, stroke, and other CVD causes.

**Figure 3.**
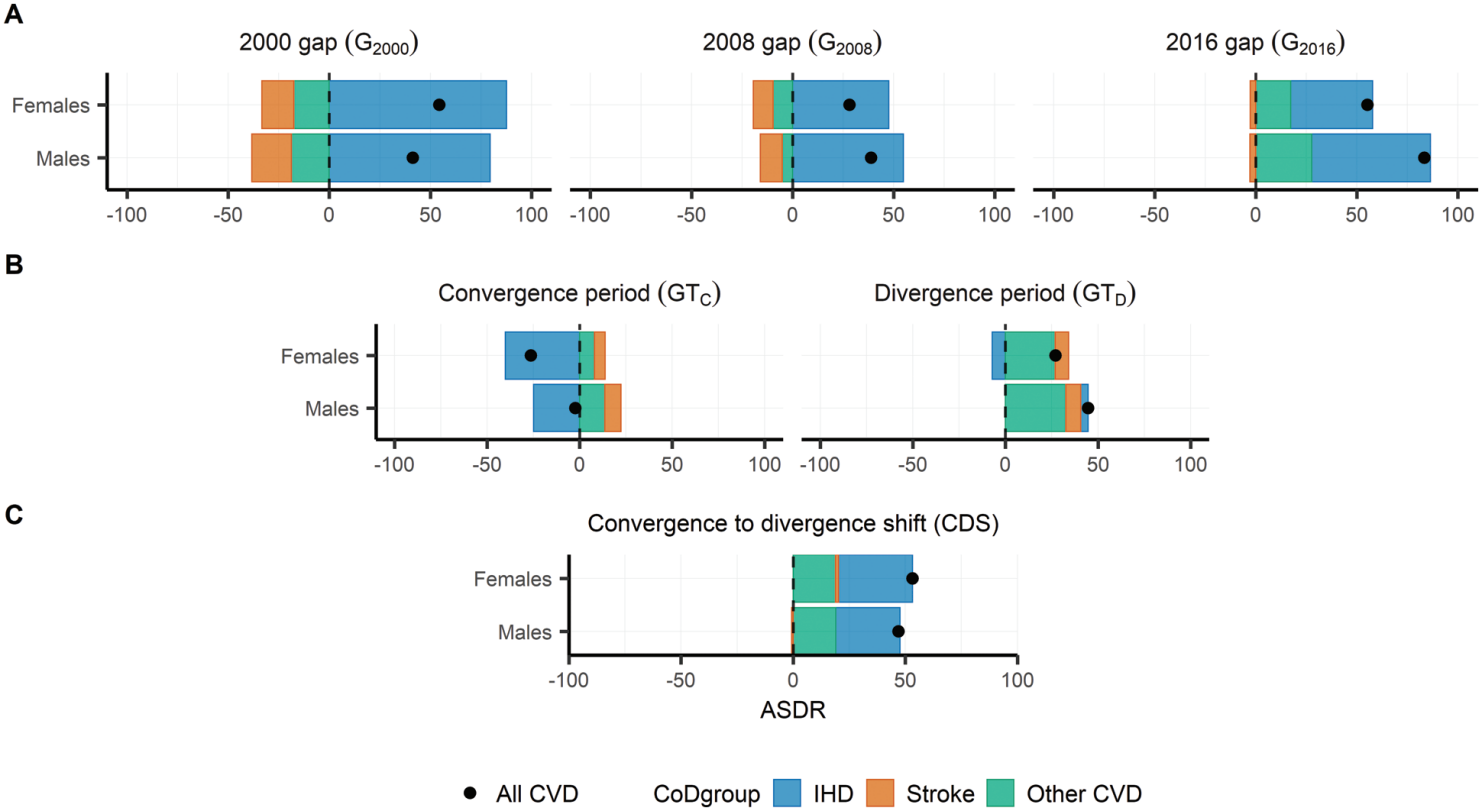
Cause-specific decomposition of overall age-standardized CVD death rate differences between the United States and other HLCs, for females and males. Panel (A) presents the decomposition of the U.S.–HLC gap in 2000, 2008, and 2016. Panel (B) presents the decomposition of the U.S.–HLC gap trend during the narrowing-gap (2000–2008) and widening-gap periods (2008–2016). Panel (C) presents the decomposition of the convergence-to-divergence shift between the narrowing-gap (2000–2008) and widening-gap periods (2008–2016). In each panel, the upper row corresponds to females and the bottom to males. The points indicate the difference in overall ASDRs of CVD mortality, and the bars the contributions of IHD, stroke, and other CVD causes to the overall CVD mortality difference. CVD = cardiovascular disease; HLCs = high life expectancy countries; ASDRs = age-standardized death rates; IHD = ischemic heart disease.

Figure 3 presents the cause-specific decomposition of the U.S.–HLC gaps (*G_t_*) in 2000, 2008, and 2016 (Panel A); the GTs during the convergence (GT_C_) and divergence (GT_D_) periods (Panel B); and the *CDS* (Panel C). The points indicate the difference in overall CVD mortality (in ASDR). The bars depict the contributions of IHD, stroke, and other CVD causes to the total CVD mortality differences. The values of these decompositions are presented in Supplementary Tables 4–6.

The decomposition of the gaps (Figure 3A), with positive values indicating higher mortality in the United States, shows that the United States had higher overall CVD mortality than the average HLC level in all 3 years. Most of the U.S. mortality disadvantage in these periods resulted from higher levels of IHD mortality. However, although the United States had lower mortality from stroke and other CVD causes than the HLC average in 2000 and 2008, this mortality advantage had disappeared by 2016.

The values shown in Figure 3B express the *GT*, which indicates how the mortality difference in Panel A changed over the periods of mortality convergence (2000–2008) and divergence (2008–2016). Whereas most of the reduction in the gap during the convergence period resulted from a substantial reduction in the IHD mortality disadvantage in the United States, the subsequent divergence resulted from two dynamics. First, the United States lost its advantage in stroke mortality (contributing to about one fifth of the increase in the gap) and experienced increases in mortality from other CVD causes (around two thirds). Second, IHD mortality reductions in the United States lost their momentum, leaving the U.S.–HLC gap in IHD virtually unchanged between 2008 and 2016. These findings are consistent with the cause-specific gaps depicted in Supplementary Figure 1.

The cause-specific contributions to the overall CVD *CDS* are presented in Figure 3C. All three CVD groups contributed to the shift from mortality convergence to mortality divergence. The largest proportion of the shift resulted from a deceleration of reductions in IHD mortality (62% in females and 59% in males). Another important contribution was the shift from a mortality advantage to a disadvantage for other CVD causes (35% and 39%). The nearly complete offsetting of the advantage in U.S. stroke mortality also contributed, albeit to a much smaller extent, to the shift from mortality convergence to divergence (2.5% and 1.5%). Note that results stratified by more fine-grained CVD groups and age are included in the supplementary materials. From these estimates, we observe that a substantial part of the CVD CDS in absolute terms was contributed by ages 85 and older, likely driven by the generally higher magnitude of mortality at those ages. Nevertheless, at least 36% (females) and 65% (males) of the overall CVD *CDS* resulted from slowing mortality decreases or even mortality increases at ages 50–84. It is noteworthy that when the U.S.–HLC gap is analyzed in relative terms, the highest disadvantage in CVD mortality during the three periods in which the gap is analyzed is at ages 25–60 (Supplementary Figure 2).

### Comparison Between CVD and Other Causes of Death

**Figure 4.**
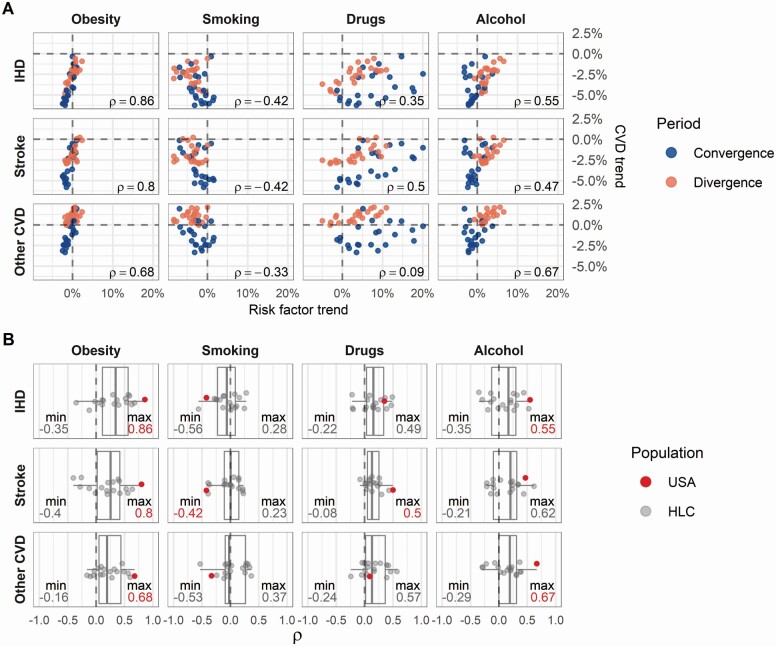
Correlation of age- and sex-specific mortality trends between CVD and risk-related causes during the convergence and divergence periods. Panel (A) plots all age- and sex-specific mortality trends during convergence and divergence periods for all combinations of CVD (rows) and risk-associated (columns) causes of death. The correlation of mortality trends between each CVD and risk-associated cause is indicated by Spearman’s correlation coefficients (ρ) at the bottom-right corner of each embedded plot. Panel (B) presents the Spearman’s correlation coefficients for the United States and other HLCs. The minimum and maximum coefficients for each CVD and risk-associated cause combination are indicated at the bottom-left and bottom-right corners of each embedded plot. When this minimum or maximum coefficient corresponds to the United States, it is depicted in red. CVD = cardiovascular disease; HLCs = high life expectancy countries; IHD = ischemic heart disease.

Figure 4A presents a scatterplot for age- and sex-specific average annual mortality changes in the United States between each CVD cause (rows) and each risk factor group (columns) during the convergence and divergence periods. The Spearman’s rank correlation coefficients are presented at the bottom-right corner of each plot in Figure 4A. The United States shows strong positive correlations between the mortality changes in the different CVD groups and those in obesity-related mortality (between 0.66 and 0.86). The correlations are slightly lower for alcohol-related mortality (between 0.45 and 0.67) and substantially lower for drug mortality (between 0.1 and 0.49). Smoking-related mortality has a negative correlation (between −0.33 and −0.45).

To put the magnitude of the U.S. correlations in context, Figure 4B shows the boxplots for the country-specific Spearman’s rank correlation coefficients for each CVD and risk factor combination. Among all 18 HLCs under observation, the United States shows the highest positive correlation between the changes in all CVD groups and obesity-related mortality, as well as between changes in IHD and other CVD and alcohol-related mortality. An almost equally extreme but opposite pattern can be observed for the correlation between changes in CVD mortality and smoking-related mortality, with the United States having one of the largest negative relationships among all observed HLCs. This means that CVD and smoking-related mortality changes moved in different directions. For drug-related mortality, the U.S. correlations are positive but not exceptionally large compared to those of other countries.

**Figure 5.**
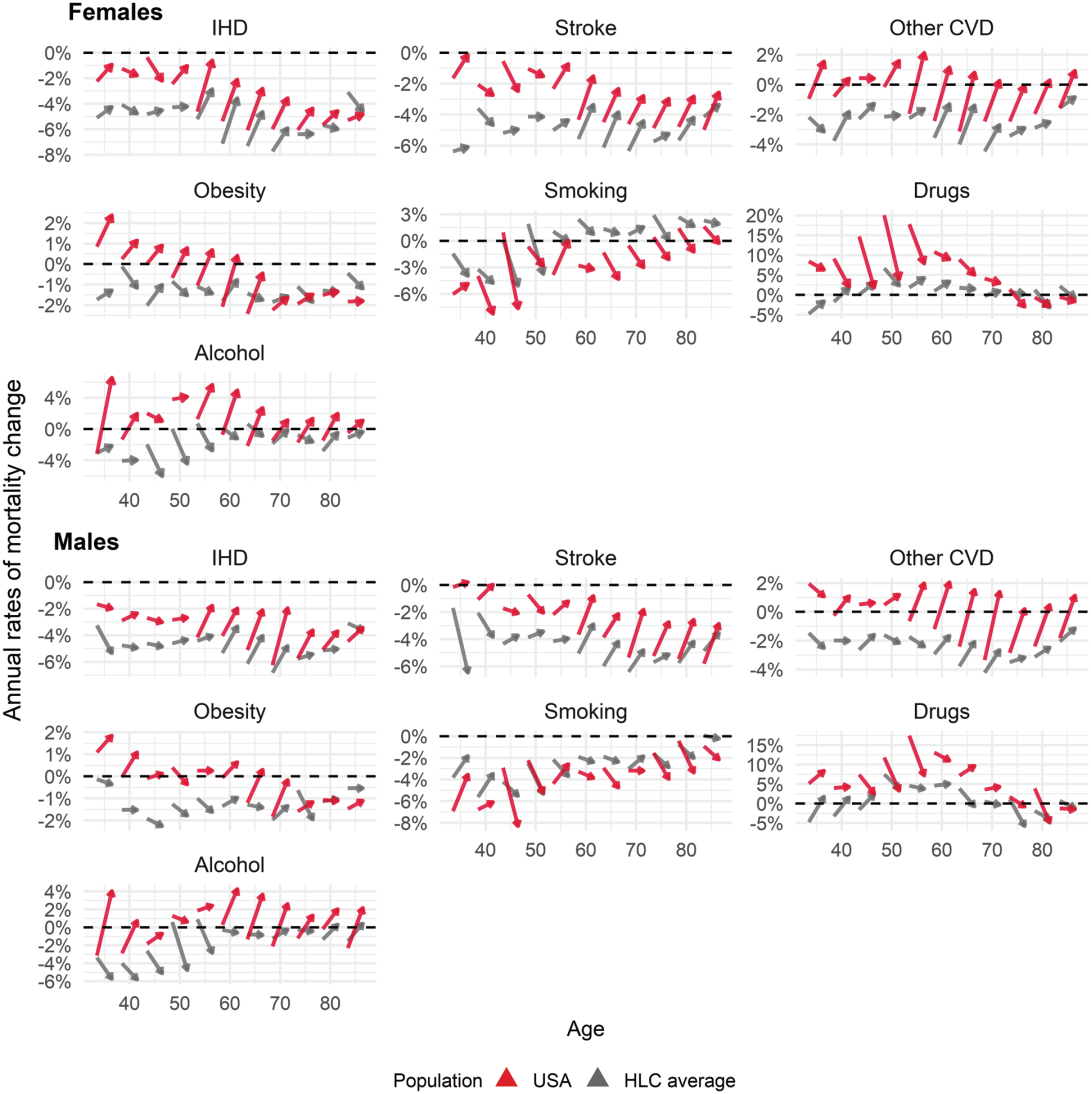
Differences in the average trends of age-specific annual changes by cause during the periods of CVD gap convergence and divergence for females (Panel A) and males (Panel B). The average annual trends are compared between the periods of gap convergence (starting point of arrows) and divergence (ending point of arrows). The convergence period is defined as 2000–2008, and the divergence period as 2008–2016. The horizontal dashed lines indicate no change in mortality (stall), and values below and above zero indicate, respectively, mortality decreases and increases. CVD = cardiovascular disease; IHD = ischemic heart disease; HLC = high life expectancy countries.

Figure 5 depicts the changes in the average annual sex- and age-specific rate of mortality change between the convergence (2000–2008) and divergence (2008–2016) periods in the United States and HLCs, separately for IHD, stroke, other CVD, obesity-related, smoking-related, drug-related, and alcohol-related mortality. The beginning of each arrow marks the average annual change during 2000–2008, and the arrowhead marks the average change during 2008–2016.

The trend change between the two periods suggests a substantial slowdown in IHD and stroke mortality reductions at all ages older than 50. Moreover, for the other CVD causes group, the trends even shifted from mortality reductions to mortality increases at all ages older than 55 for both sexes in the United States. Although reductions also slowed for the HLC average, the magnitude of this deceleration was noticeably smaller than that for the United States.

Two further observations emerge when comparing the trend changes of CVD mortality to those of risk factor–related groups. First, the age-specific patterns of trend changes in CVD causes and obesity- and alcohol-related mortality are very similar. For all CVD causes and obesity- and alcohol-related mortality, there was either a slowdown in reductions, a shift from decreasing to increasing mortality, or even an acceleration of preexisting mortality increases. Note that the above-mentioned trends in obesity- and alcohol-related mortality in the United States changed inversely to those in the HLC average. Second, smoking- and drug-related mortality trend changes followed the opposite pattern of those of CVD causes. Whereas declines in smoking-related mortality accelerated during the divergence period, increases in drug-related mortality continued, but slowed considerably.

## Discussion

Previous research documented that U.S. CVD mortality has almost stopped declining since around 2010 (Lopez & Adair, 2019; Mehta et al., 2020). In this study, we used information on 18 HLCs, including the United States, and investigated the underlying mortality dynamics and the potential drivers of this trend change. Our analysis generated four key findings that demonstrate the exceptionalism of the U.S. CVD mortality trend. *First*, U.S. CVD mortality is considerably higher than the average of other HLCs. *Second*, the gap between the United States and the HLC average CVD mortality declined until around 2008 and increased thereafter. Specifically, U.S. female and male CVD mortality decreased faster than that in almost half of the analyzed HLC—including countries with steady decreases, like Japan or France—between 2000 and 2008, but decreased very little in the following years; whereas most HLC experienced continued reductions in CVD mortality, albeit at a slower pace than in the first period. *Third,* the shift from convergence to divergence was mainly driven by both slowing IHD and stroke mortality declines and increasing mortality from other CVD causes. *Fourth*, among the observed risk-related causes of death, only the trend changes in obesity- and alcohol-related mortality had age and temporal patterns that were similar to those observed for CVD mortality in the United States.

### The Temporal Dynamics and the Cause-Specific Contributions to the Stagnation in CVD Mortality

The U.S. penalty in overall CVD mortality resulted from unfavorable changes for all CVD causes. This suggests that the stalling of the decline in CVD mortality cannot be attributed to changes in a single CVD, particular medical treatments or diagnoses, or the influence of a single extrinsic factor. In line with previous findings on rising mortality among the working-age population in the United States, our results suggest that gradual changes in the general health status of large shares of the population are the key drivers of this development (NASEM, 2013, 2021). These dynamics have already and will likely continue to have detrimental effects on mortality from causes other than CVD. Thus, the factors driving the current adverse mortality trend also have broad implications for the general long-run development of U.S. mortality.

Nearly two thirds of the widening gap in CVD mortality after 2008 stemmed from slowing mortality reductions or even mortality increases at ages 55–84 (Supplementary Figure 4). The magnitude of these contributions was considerable given that deaths from CVD occur disproportionately at older ages (Ocaña-Riola et al., 2015; Okui, 2020; Parkinson et al., 2020). However, the CVD mortality penalty at middle ages, for which the largest relative gap was found (Supplementary Figure 2), also points to opportunities for further mortality reductions, and, thus, for increases in U.S. life expectancy.

We also found that the oldest age group in our analysis (85+) contributed considerably to the CVD mortality divergence, which is noteworthy given that the United States performs well in old-age mortality (Preston & Vierboom, 2021). However, this large contribution is not surprising given that death rates are highest at ages 85 and older, and thus a small relative change at those ages can result in much higher absolute effects than at younger ages. For CVD, this is likely the case at ages 85 and older. Despite the methodological considerations, our results still suggest that given the mortality developments in this age group, the United States is progressively losing its previous advantage in old-age mortality relative to other countries. A possible explanation for this shift is that U.S. cohorts with health disadvantages, such as the boomers, are entering old ages.

### The Role of Risk Factors

We found similarities in the trends and trend changes between CVD mortality and mortality from causes related to alcohol abuse and obesity, while we observed the opposite dynamics for smoking- and drug-related mortality. This suggests that alcohol abuse and obesity might be key factors in the stalling of CVD mortality declines in the United States.

The links between obesity and alcohol intake and an elevated risk of developing different CVDs have been extensively documented (Bell et al., 2017; Di Cesare et al., 2013; Klatsky, 2015; Stokes & Preston, 2017; Wood et al., 2018). Despite the general exacerbating effects of both factors, the age pattern of mortality change is especially concerning when considering future trends in CVD mortality. The large contribution of mortality at young ages to mortality increases suggests that the health burden of obesity and alcohol abuse will continue to increase in the future and may have even stronger adverse effects. As both mechanisms tend to accumulate and worsen over the life course, their adverse effects on CVD mortality will be increasingly magnified at older ages.

In line with this pattern, previous research found a gradual increase in obesity prevalence and obesity-attributable mortality at younger ages across birth cohorts (Masters et al., 2013). Thus, increasing shares of more recent birth cohorts are exposed to the adverse effects of obesity earlier in life and for a longer time. It therefore appears that rising obesity prevalence and obesity-related mortality are general trends, rather than transient health burdens specific to certain birth cohorts.

For alcohol-attributable mortality, increases are also observed at most ages (Spillane et al., 2020; Vierboom, 2020; White et al., 2020). However, unlike obesity trends, increases in alcohol-related mortality are particularly driven by the baby boomer and millennial birth cohorts (Duncan et al., 2010; Keyes & Miech, 2013; Rao & Roche, 2017; Spillane et al., 2020). There is evidence that these cohorts exhibit general excess mortality for several behavior-related causes of death, including drug- and alcohol-related causes (Acosta et al., 2020). Hence, for these cohorts, the adverse effects of obesity may be additionally modulated by unhealthy behaviors like alcohol abuse, which could eventually cause the particularly large increases in CVD mortality that we found for the respective ages.

Changes in smoking prevalence have also been discussed as a potential cause for the deceleration in CVD mortality reductions (Lopez & Adair, 2019; Mehta et al., 2020). As the prevalence of smoking has reached low levels in the United States, it has been hypothesized that only small additional gains are possible, and, thus, that the decline in smoking has lost its strength as a driver of CVD mortality reductions. However, our findings do not support this hypothesis and instead demonstrate the opposite pattern. During the period in which decreases in CVD mortality decelerated in the United States, decreases in smoking-related mortality accelerated. Moreover, the decrease in the rate of CVD mortality reductions was more pronounced at ages that also had the greatest acceleration in smoking mortality reductions (Figures 3 and 4 and Supplementary Figures 5 and 6). The change in smoking-related mortality may even suggest that smoking is still positively contributing to CVD mortality reductions or is at least counterbalancing the increases.

An alternative mechanism that could modulate the stalling of CVD reductions is the ongoing opioid epidemic in the United States. Our findings do not support this hypothesis because U.S. CVD mortality stayed almost constant or slowed down the most at the ages with the strongest deceleration in drug-related mortality increases. Nevertheless, while drug-related mortality may not be the main driver of the stagnation in CVD mortality, it remains high and may therefore have contributed to the slowdown in reductions.

### Limitations

Miscoding and misclassification are among the most common limitations of cause-of-death analyses. To acknowledge this problem, we used only broad CVD groups. Although deaths from more specific causes, such as hypertension and heart failure, have been found to account for the greatest increases in premature deaths in the United States between 2011 and 2018 (Shah et al., 2020), different studies have suggested that many of these deaths were actually caused by IHD or stroke (Di Cesare et al., 2013; Lozano et al., 2001; Naghavi et al., 2010). We thus decided to present estimates including more detailed CVD causes in the supplementary materials only.

We were not able to control for the potential sources of distortion that may be particularly problematic in cross-country comparisons (Lozano et al., 2001; Pagidipati & Gaziano, 2013). Although differences in classification may lead to nonnegligible biases, it is noteworthy that such distortions mainly affect level differences between countries, but are less likely to distort changes in the temporal trends within the same country (Lopez & Adair, 2019), as well as changes in the U.S.–HLC GTs.

It is, however, clear that this approach neglects specific deviating trends, such as those in Austria, Finland, Germany, or Japan; and it fails to take into account that country-specific convergence–divergence breakpoints may differ from those observed for the average. Nonetheless, we attempted to investigate U.S. exceptionalism by comparing the United States with a general international trend, rather than comparing it with specific countries. For our purposes, using the HLC average as a comparison group was a reasonable simplification; and although a country-specific comparison would be informative, this level of analysis was beyond the scope of this article.

Our use of cause-specific mortality to indicate the effect of a certain risk factor is another limitation. Previous studies that used more sophisticated methods, such as population-attributable fractions, to quantify the mortality impact of diabetes or alcohol consumption found considerably higher mortality shares than studies that used only underlying causes of death (Stokes & Preston, 2017; Trias-Llimós et al., 2018). As these types of biases certainly also apply to our risk factor–related mortality estimates, the results should be interpreted with caution. However, we did not aim to provide precise estimates of the total mortality toll. Instead, we sought to analyze and compare the patterns of mortality change, which we found to be consistent with those published in earlier work that used a different methodology.

Another issue that could arise because our analysis did not consider any socioeconomic dimensions is a potential distortion of the U.S. CVD mortality trends by the Affordable Care Act (ACA). As a consequence of the ACA, a large share of presumably less healthy subpopulations received health insurance coverage, particularly between 2013 and 2015 (Courtemanche et al., 2019). This may have improved the precision of individual medical records, diagnoses, and death certificates, which might, in turn, have affected cause-of-death classification and thus the share of CVD deaths.

## Conclusion

Our analysis demonstrated the exceptionalism of the development of U.S. CVD mortality compared to that in most other HLCs. Although most HLCs also experienced a slowdown in CVD mortality, the stall observed for the United States stands out from the general pattern. The exceptional CVD mortality changes in the United States are driven by a distinct pattern of a deceleration of reductions in IHD and stroke mortality and increases in mortality from other CVD causes. The emergence of this pattern suggests that a rising population health burden is a key driver. Changes in CVD mortality correlate strongly with obesity- and alcohol-related mortality, with the latter being particularly pronounced in specific birth cohorts. This suggests that an important share of the CVD mortality stagnation may be driven by obesity, with alcohol abuse potentially being an additional modulating factor. There is a need for further research analyzing whether widening inequalities within the U.S. population represent a potential systemic factor affecting several health and mortality outcomes.

## Supplementary Material

gbac032_suppl_Supplementary_Materials
