# Supplementary material for: Cardiovascular Mortality Gap Between the United States and Other High Life Expectancy Countries in 2000–2016
Source: J Gerontol B Psychol Sci Soc Sci. 2022 Feb 23;77(Suppl 2):S148–57. doi: 10.1093/geronb/gbac032 (PMC9154236; doi:10.1093/geronb/gbac032)
Supplement: gbac032_suppl_Supplementary_Materials [file gbac032_suppl_supplementary_materials.pdf]

Supplementary materials: Cardiovascular mortality gap between the US and other high life expectancy countries in 2000-2016

Countries included in the analyses

Table S1 presents the list of countries included in the analysis and the period (in parenthesis) in which mortality by cause of death classified using ICD-10 codes are available.

Table S1. High-life expectancy countries and observation periods (in parenthesis) included in the analyses.

USA (2000-2016)	Belgium (2000-2016)	Finland (2000-2016)	Italy (2003-2016)	New Zealand (2000-2016)	Sweden (2000-2016)
Australia (2000-2004, 2006-2016)	Canada (2000-2016)	France (2000-2016)	Japan (2000-2016)	Norway (2000-2016)	Switzerland (2000-2016)
Austria (2002-2016)	Denmark (2000-2016)	Germany (2000-2016)	Netherlands (2000-2016)	Spain (2000-2016)	UK (2001-2016)

Causes of death

Table S2 lists the ICD-10 codes that were used to identify deaths from the selected causes under observation.

Table S2. Causes of deaths and ICD-10 Codes

**Causes of death**		**ICD-10 codes**
Ischemic heart diseases (IHD)		I20-25
Stroke		I61-64
Other cardiovascular diseases	Hypertensive diseases	I10-15
	Heart failure	I50
	Other Heart diseases	I00-09; I26-49; I51-52
	Other cerebrovascular diseases	I60, I65-69
	Circulatory diseases	I70-99
Obesity-related mortality	Obesity cancer-related diseases	C15-16; C18-25
	Obesity and diabetes	E10-14; E66
Smoking-related mortality		C33-34; J44:47
Alcohol-related mortality		F10; K70; K73-74; X45
Drug-related mortality		F11-19; F55; X40-44; Y10-14; T40

Changes in CVD mortality improvements during the convergence and divergence period

Table S3 presents the trends of CVD mortality (measured in annual percentage change of ASDR) during the periods 2000-2008 (second column) and 2008-2016 (third column), and the trend changes (fourth column), for the 18 countries under analysis and HLC average (excluding the US).

Table S3. Trends in ASDR of CVD during the periods 2000-2008 and 2008-2016

Country	CVD average trend (ASDR) in %		Trend change	Year with the minimum gap relative to the US
	2000-2008	2008-2016		
**USA**	**-3.91**	**-1.52**	**-61.16**	**-**
Austria	-4.21	-1.94	-53.94	2002
Canada	-4.35	-2.66	-38.89	2009
Netherlands	-4.40	-2.78	-36.77	2004
Italy	-4.00	-2.57	-35.71	2009
Norway	-4.69	-3.85	-17.81	2000
Belgium	-3.85	-3.28	-14.85	2004
**Average**	**-3.78**	**-3.17**	**-14.12**	**2009**
UK	-4.97	-4.30	-13.52	2001
New Zealand	-3.39	-2.98	-12.11	2009
France	-3.55	-3.19	-10.08	2010

Country	CVD average trend (ASDR) in %		Trend change	Year with the minimum gap relative to the US
	2000-2008	2008-2016		
Denmark	-4.80	-4.42	-7.90	2002
Australia	-4.07	-3.77	-7.28	2008
Spain	-3.23	-3.02	-6.38	2009
Sweden	-3.13	-2.96	-5.56	2009
Switzerland	-3.31	-3.25	-1.75	2009
Finland	-2.98	-3.02	1.62	2009
Germany	-2.95	-3.04	2.99	2004
Japan	-2.46	-2.90	17.88	2011

Trends in the US-HLE Gap by CVD cause and sex

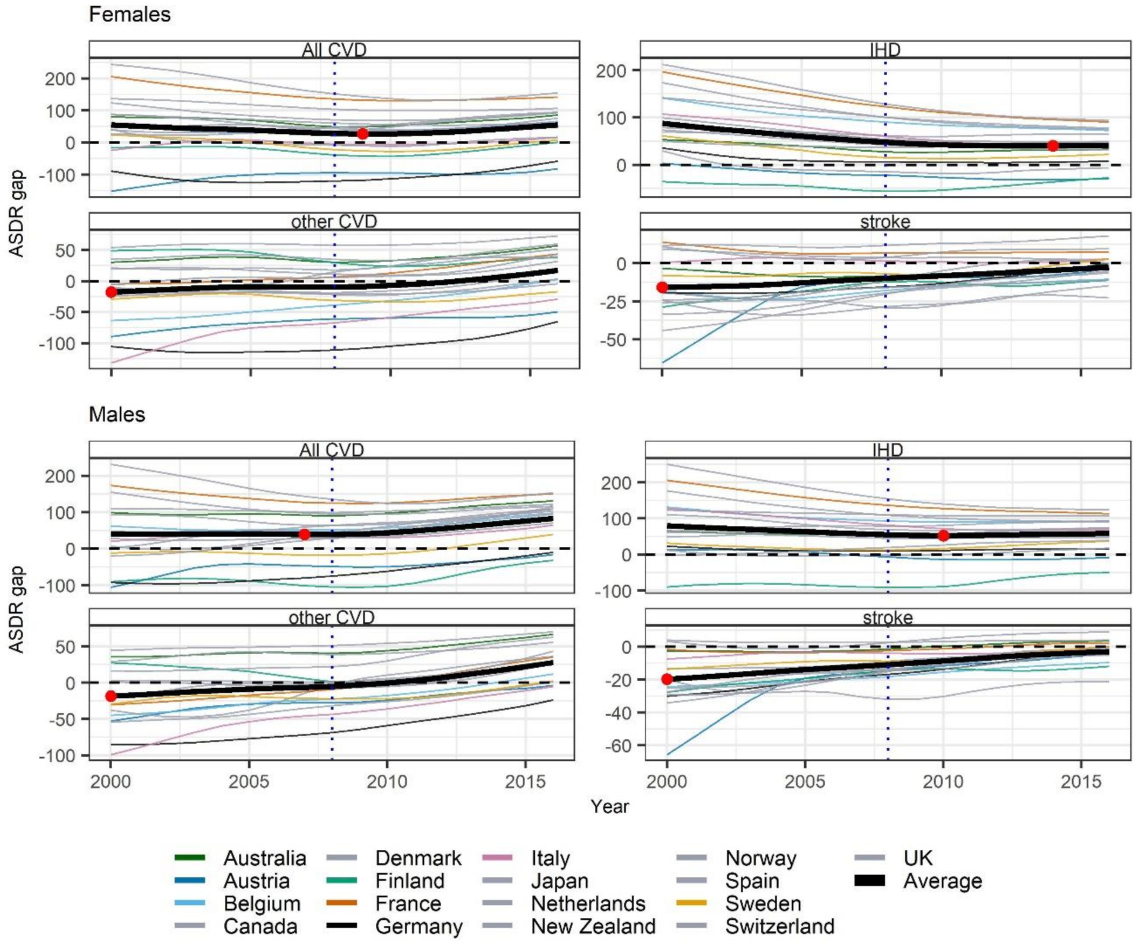

Figure S1. US-HLC gap in age-standardized death rates from CVD causes by sex. The US-HLC average gap is indicated with a thick black line and the gap with each HLC with thinner colored and grey lines. The red dot indicates the year in which the US-HLE gap attained the minimum magnitude during the observation period. The dotted vertical line indicates the year 2008 when the US-HLC gap shifted from convergence to divergence. The US population in 2016 is used as the reference.

Relative US and HLC gap in CVD mortality

Figure S2 presents the US-HLC gap by age and sex in the years 2000, 2008, and 2016.

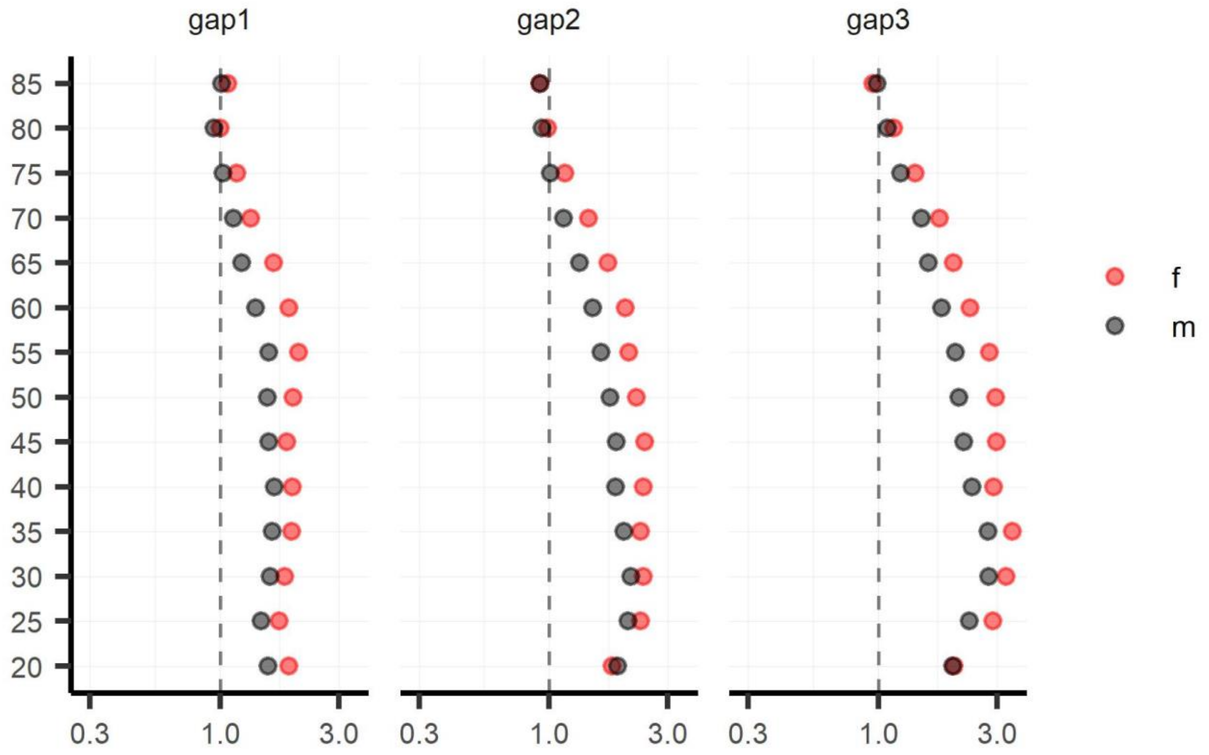

Figure S2. Age- and sex-specific relative CVD gap between the US and HLC

Decomposition results based on an alternative grouping of the CVD group

Figure S3 presents the cause-specific decomposition of the US-HLC gap trends in CVD mortality among seven CDV groups. Panel (A) depicts the cause-specific decomposition of the gap in the years 2000, 2008, and 2016, Panel (B) the decomposition of the narrowing- and widening-gap trends, and Panel (C) the decomposition of the convergence-to-divergence shift.

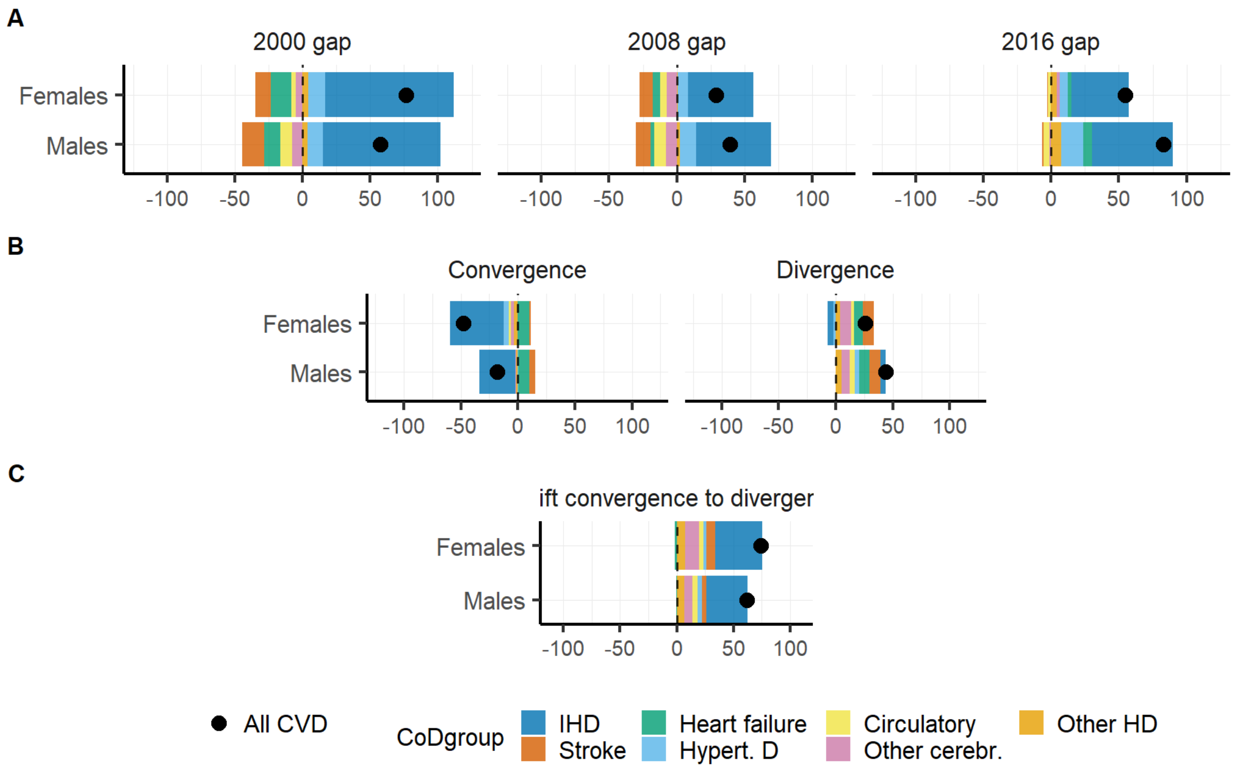

Figure S3. Cause-specific decomposition of overall age-standardized CVD death rates differences between the US and other HLC, for females and males. **Panel (A)** presents the decomposition of the US-HLC gap in periods 2000, 2008, and 2016; **Panel (B)** presents the decomposition of the US-HLC gap trend during the narrowing- (2000-2008) and widening-gap periods (2008-2016). **Panel (C)** presents the decomposition of the convergence-to-divergence shift between the narrowing- (2000-2008) and widening-gap periods (2008-2016). In each panel, the upper row corresponds to females and the bottom to males. The points indicate the difference in overall ASDR of CVD mortality, and the bars the contribution of seven CVD causes to the overall CVD mortality difference.

Table S4. Decomposition of the US-HLC gap in CVD mortality during the periods 2000, 2008, and 2016. Units are in age-standardized death rates (ASDR), using US population in 2016 as the reference.

	Sex	CoDgroup	G2000		G2008		G2016	
			Cont	Total	Cont	Total	Cont	Total
Gap (G)	Females	Circulatory	-4.74	54.44	-4.74	28.21	-2.70	55.20
		Heart failure	-12.65		-5.61		5.61	
		Hypert. D	11.06		7.93		6.67	
		IHD	87.73		47.62		40.55	
		Other cerebr.	-9.64		-7.68		2.35	
		Other HD	-1.56		0.55		5.38	
		Stroke	-15.76		-9.87		-2.66	
	Males	Circulatory	-9.72	41.26	-8.84	38.89	-4.39	83.48
		Heart failure	-9.75		-2.54		8.12	
		Hypert. D	10.54		11.85		16.32	
		IHD	79.63		54.86		58.72	
		Other cerebr.	-10.19		-7.84		-0.54	
		Other HD	0.44		2.33		8.13	
		Stroke	-19.70		-10.94		-2.89	

Table S5. Decomposition of the US-HLC gap trend during the narrowing-gap (2000-2008) and widening-gap periods (2008-2016). Units are in age-standardized death rates (ASDR), using US population in 2016 as the reference.

	Sex	CoDgroup	GT 2000-2008		GT 2008-2016	
			Cont	Total	Cont	Total
Gap Trend (GT)	Females	Circulatory	0.00	-26.23	2.04	26.99
		Heart failure	7.04		11.22	
		Hypert. D	-3.13		-1.26	
		IHD	-40.11		-7.06	
		Other cerebr.	1.96		10.02	
		Other HD	2.11		4.83	
		Stroke	5.89		7.21	
	Males	Circulatory	0.87	-2.37	4.46	44.60
		Heart failure	7.21		10.66	
		Hypert. D	1.31		4.47	
		IHD	-24.77		3.86	
		Other cerebr.	2.35		7.30	
		Other HD	1.89		5.80	
		Stroke	8.76		8.05	

Table S6. Decomposition of the convergence-to-divergence shift between the narrowing-gap (2000-2008) and widening-gap periods (2008-2016). Units are in age-standardized death rates (ASDR), using US population in 2016 as the reference.

	Sex	CoDgroup	CDS	
			Cont	Total
Convergence-to-Divergence Shift (CDS)	Females	Circulatory	2.04	53.22
		Heart failure	4.18	
		Hypert. D	1.86	
		IHD	33.05	
		Other cerebr.	8.06	
		Other HD	2.71	
		Stroke	1.32	
	Males	Circulatory	3.58	46.97
		Heart failure	3.45	
		Hypert. D	3.16	
		IHD	28.64	
		Other cerebr.	4.95	
		Other HD	3.91	
		Stroke	-0.71	

Figure S4 presents the age- and cause-specific decomposition of the convergence-to-divergence shift into three CVD causes for each sex.

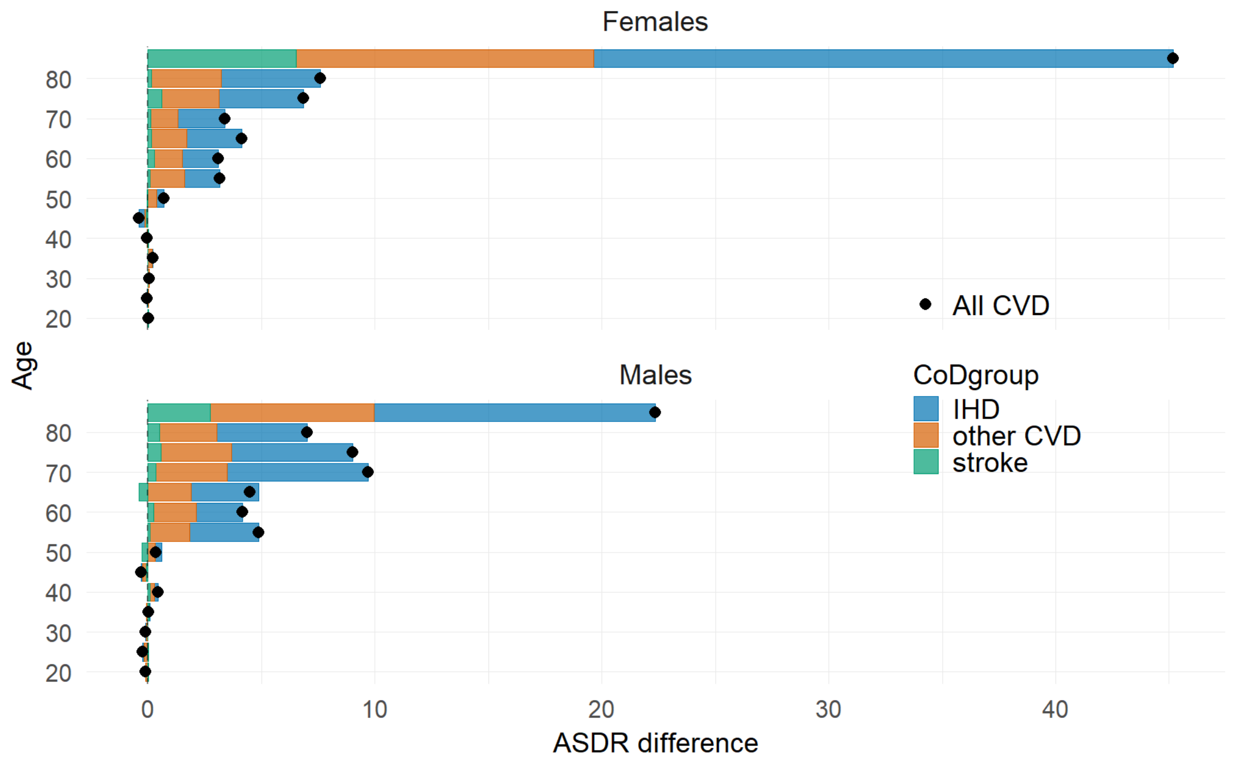

Figure S4. Age- and cause-specific decomposition of the convergence-to-divergence shift between the narrowing- (2000-2008) and widening-gap periods (2008-2016). The upper row corresponds to females and the bottom to males. The points indicate the difference in overall ASDR of CVD mortality, and the bars the contribution of IHD, stroke, and other CVD causes to the overall CVD mortality difference.

Results for the analysis of CVD mortality and risk-factors-related mortality using alternative cause-of-death groupings

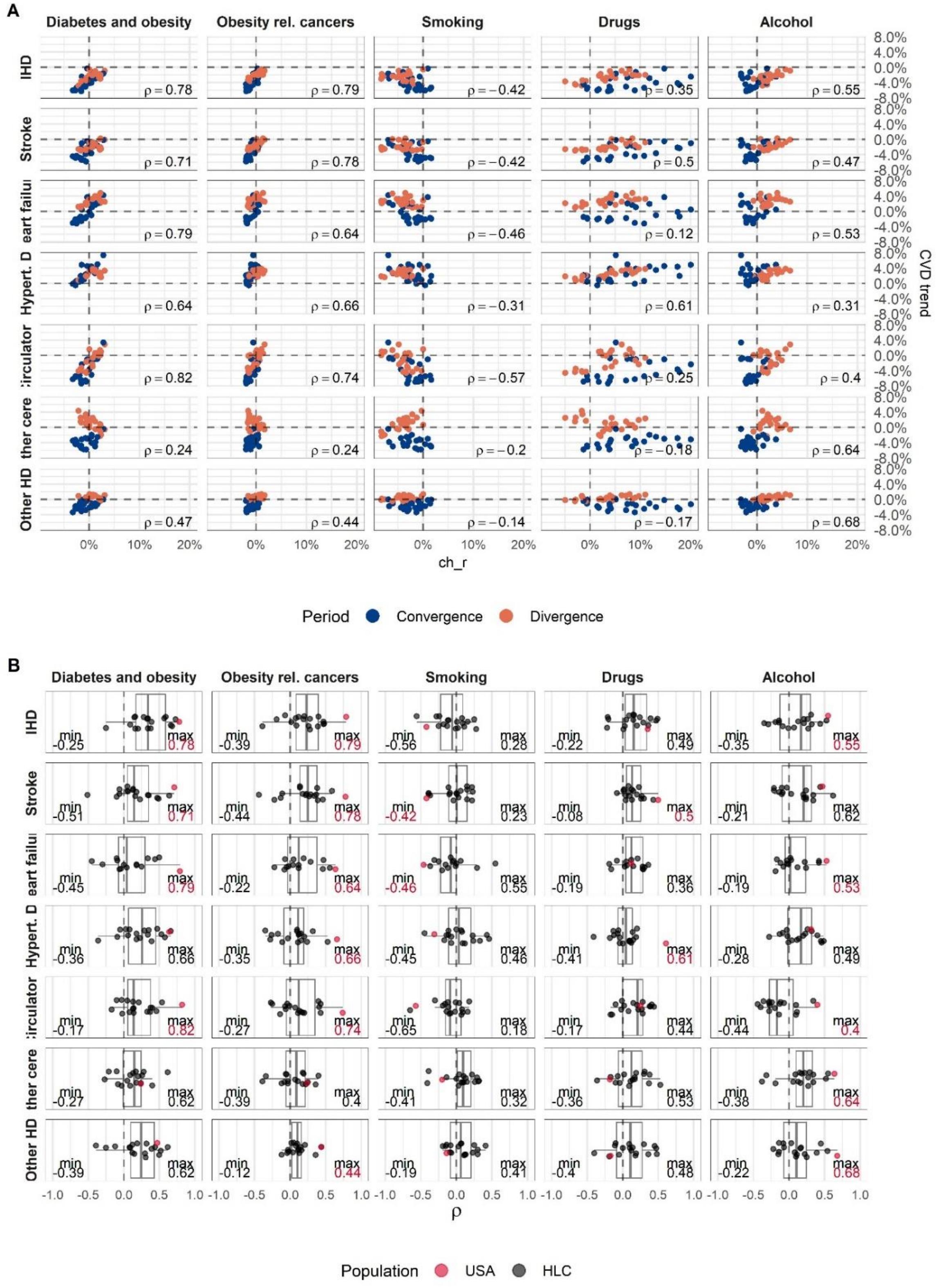

Figure S5. Correlation of age- and sex-specific mortality trends between CVD and risk-related causes during the convergence and divergence periods. Panel (A) plots all age- and sex-specific mortality trends during convergence (in blue) and divergence (in orange) periods for all combinations of CVD (rows) and risk-associated (columns) causes of death. The correlation of mortality trends between each CVD and the risk-associated cause is indicated by Spearman correlation coefficients (ρ) at the bottom right corner of each embedded plot. **Panel (B)** presents the Spearman correlation coefficients for the US (in red) and other HLC (in black). The minimum and maximum coefficients for each CVD and risk-associated cause combination are indicated at the bottom left and right corners of each embedded plot. When this minimum or maximum coefficient corresponds to the US, it is depicted in red.

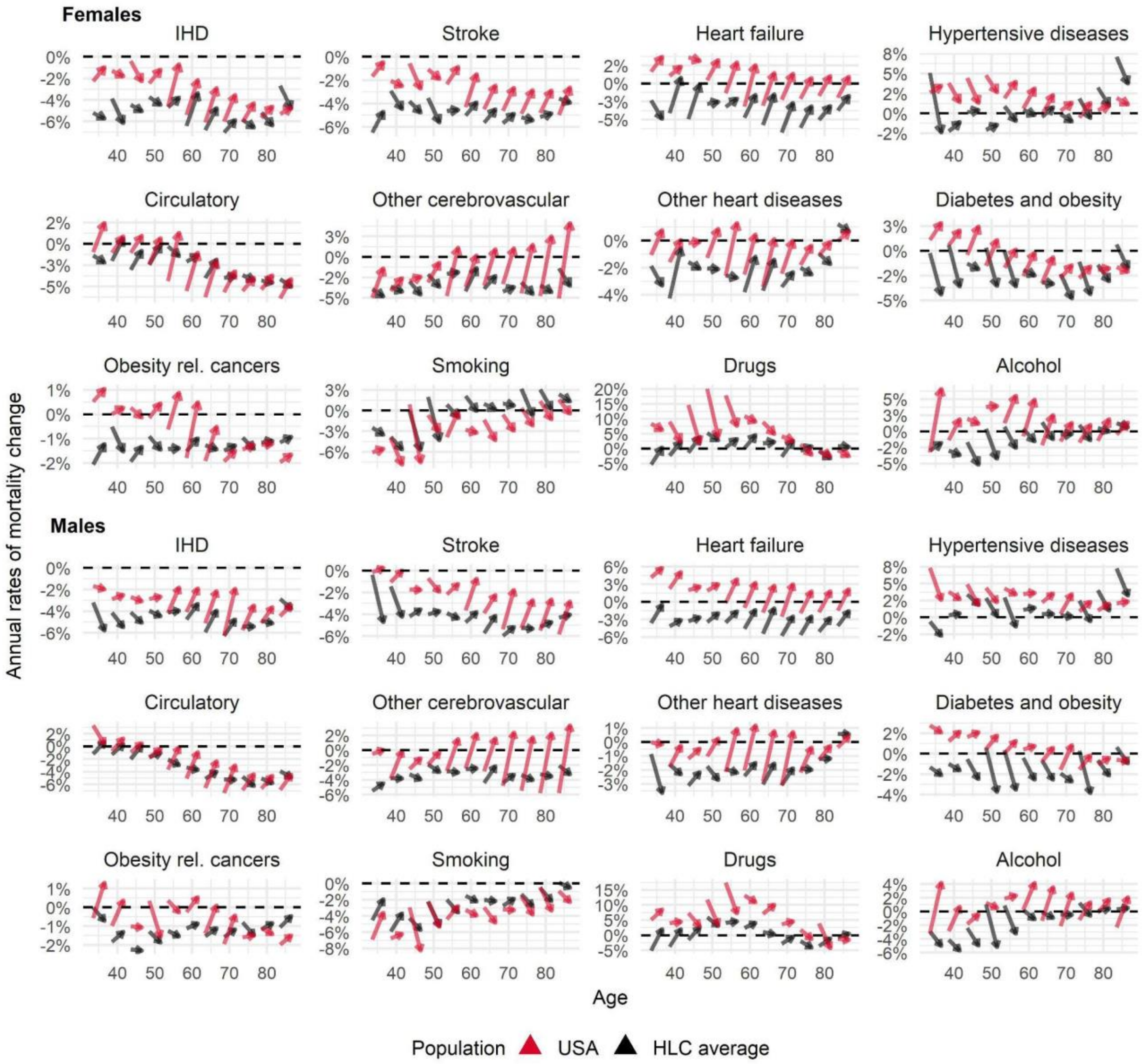

Figure S6. Differences in the average trends of age-specific annual change by cause during the periods of CVD gap convergence and divergence for females (Panel A) and males (Panel B). The average annual trends are compared between the periods of gap convergence (starting point of arrows) and divergence (ending point of arrows). The period of convergence is defined as 2000-2008, and the period of divergence as 2008-2016. The horizontal dashed lines indicate no change in mortality (stagnation), and values below and above zero indicate, respectively, mortality improvements and deterioration.

Sensitivity analyses using original data and excluding countries with incomplete data series

Robustness check 1: Estimates using original data, excluding years with missing data

Figures S7-S10 reproduce the gap trend analyses but excluding years with missing data.

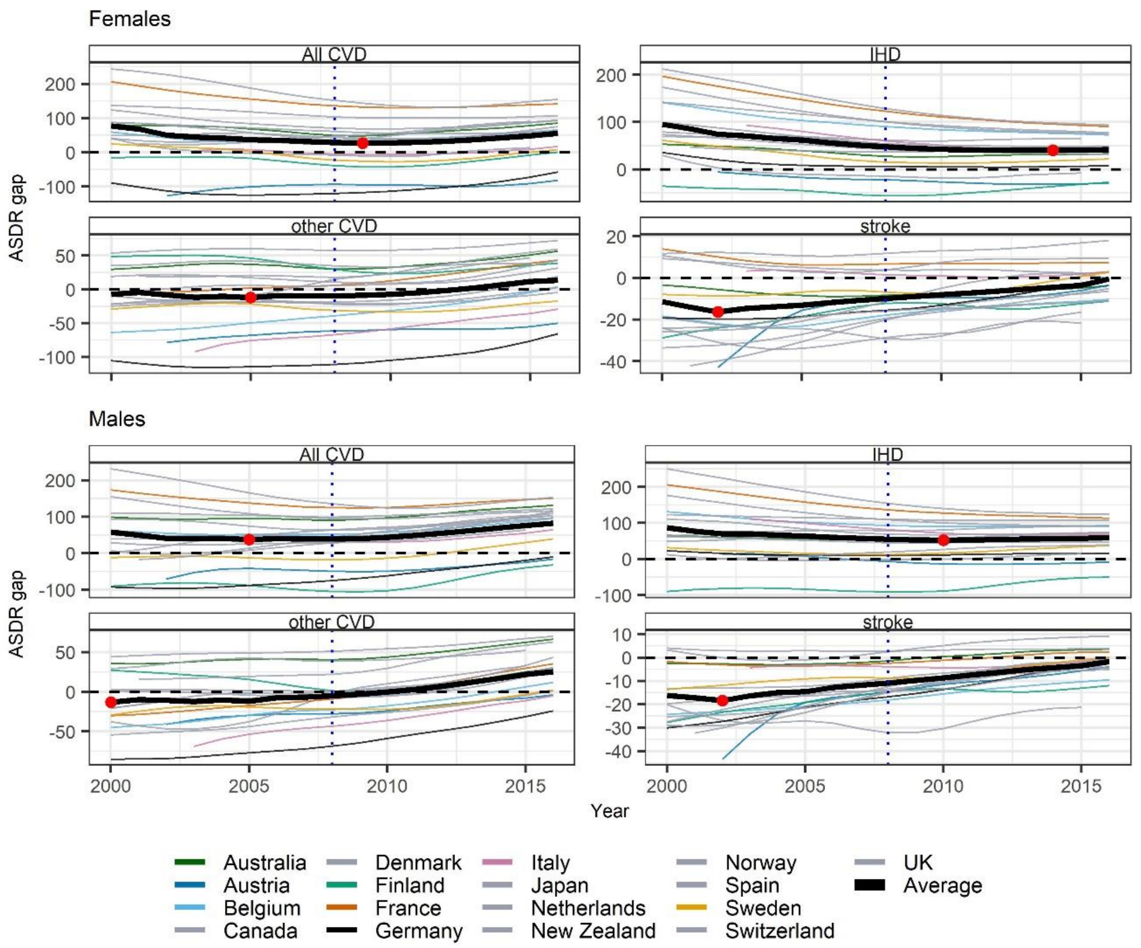

Figure S7. US-HLC gap in age-standardized death rates from CVD causes by sex, excluding years with missing data. The same description as in the Figure S1 caption applies.

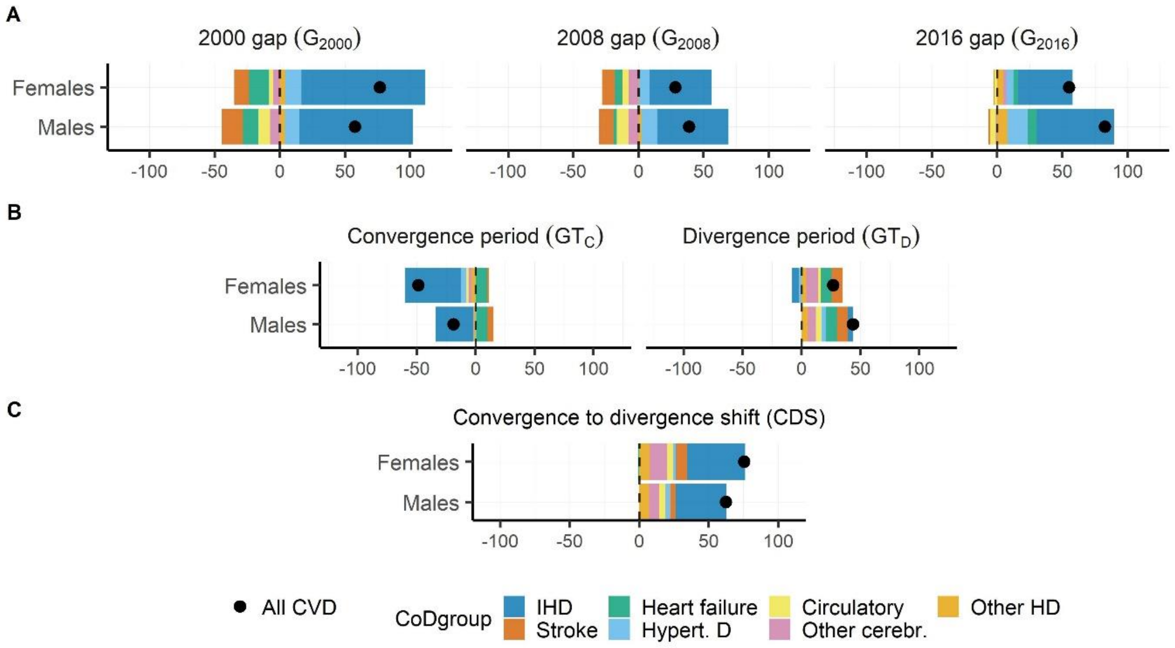

Figure S8. Cause-specific decomposition of overall age-standardized CVD death rates differences between the US and other HLC, excluding years with missing data. The same description as in the Figure S3 caption applies.

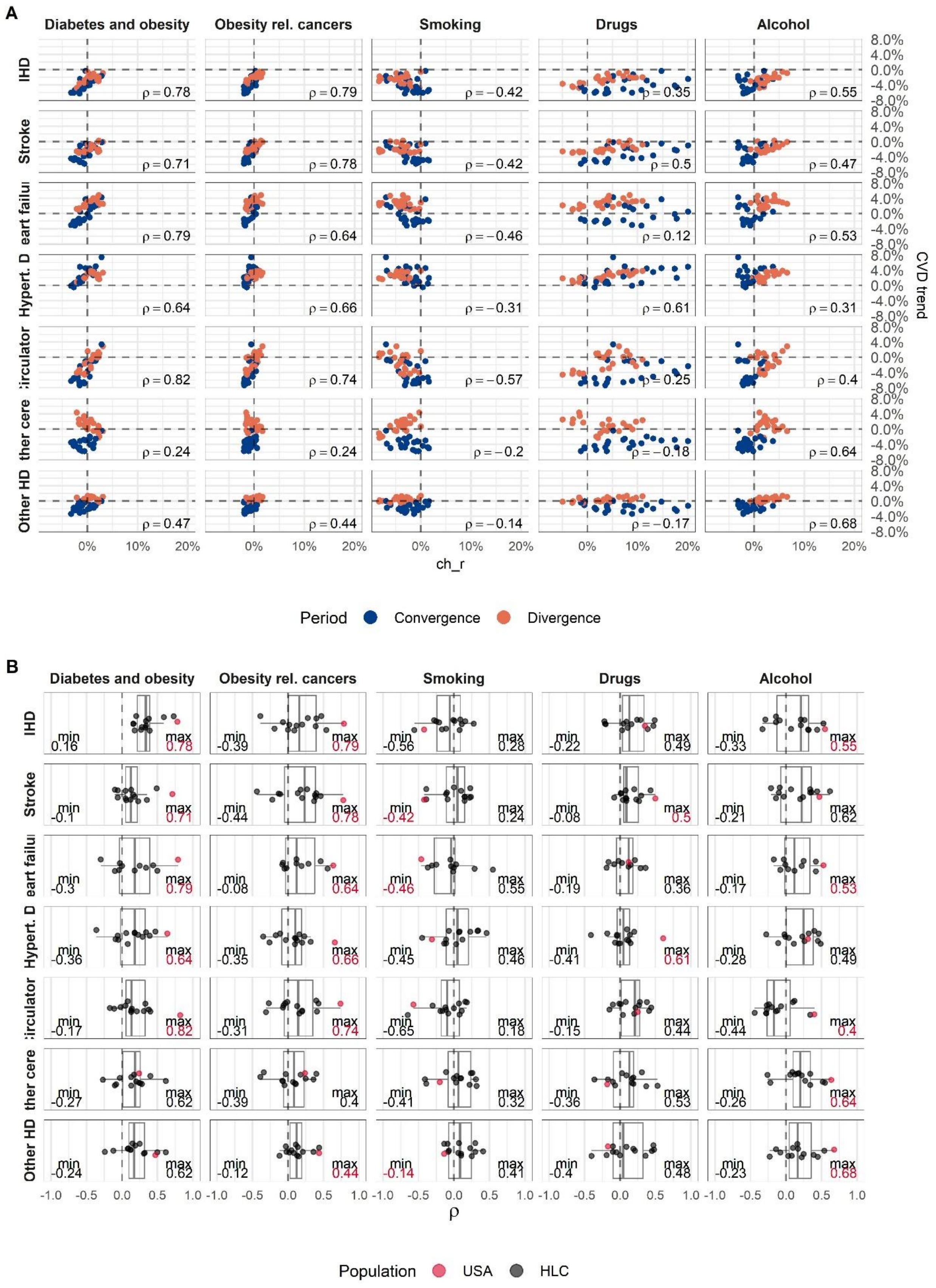
A
Diabetes and obesity
Obesity rel. cancers
Smoking
Drugs
Alcohol
IHD
Stroke
eart failu
Hypert. D
ïrculator
ther cere
Other HD
ρ = 0.78
ρ = 0.79
ρ = −0.42
ρ = 0.35
ρ = 0.55
ρ = 0.71
ρ = 0.78
ρ = −0.42
ρ = 0.5
ρ = 0.47
ρ = 0.79
ρ = 0.64
ρ = −0.46
ρ = 0.12
ρ = 0.53
ρ = 0.64
ρ = 0.66
ρ = −0.31
ρ = 0.61
ρ = 0.31
ρ = 0.82
ρ = 0.74
ρ = −0.57
ρ = 0.25
ρ = 0.4
ρ = 0.24
ρ = 0.24
ρ = −0.2
ρ = −0.18
ρ = 0.64
ρ = 0.47
ρ = 0.44
ρ = −0.14
ρ = −0.17
ρ = 0.68
8.0% 4.0% 0.0% -4.0% -8.0%
CVD trend
0% 10% 20%
ch_r
Period
Convergence
Divergence
B
Diabetes and obesity
Obesity rel. cancers
Smoking
Drugs
Alcohol
IHD
min 0.16 max 0.78
min -0.39 max 0.79
min -0.56 max 0.28
min -0.22 max 0.49
min -0.33 max 0.55
Stroke
min -0.1 max 0.71
min -0.44 max 0.78
min -0.42 max 0.24
min -0.08 max 0.5
min -0.21 max 0.62
eart failu
min -0.3 max 0.79
min -0.08 max 0.64
min -0.46 max 0.55
min -0.19 max 0.36
min -0.17 max 0.53
Hypert. D
min -0.36 max 0.64
min -0.35 max 0.66
min -0.45 max 0.46
min -0.41 max 0.61
min -0.28 max 0.49
ïrculator
min -0.17 max 0.82
min -0.31 max 0.74
min -0.65 max 0.18
min -0.15 max 0.44
min -0.44 max 0.4
ther cere
min -0.27 max 0.62
min -0.39 max 0.4
min -0.41 max 0.32
min -0.36 max 0.53
min -0.26 max 0.64
Other HD
min -0.24 max 0.62
min -0.12 max 0.44
min -0.14 max 0.41
min -0.4 max 0.48
min -0.23 max 0.68
-1.0 -0.5 0.0 0.5 1.0
ρ
Population
USA
HLC

Figure S9. Correlation of age- and sex-specific mortality trends between CVD and risk-related causes during the convergence and divergence periods, excluding years with missing data. The same description as in the Figure S5 caption applies.

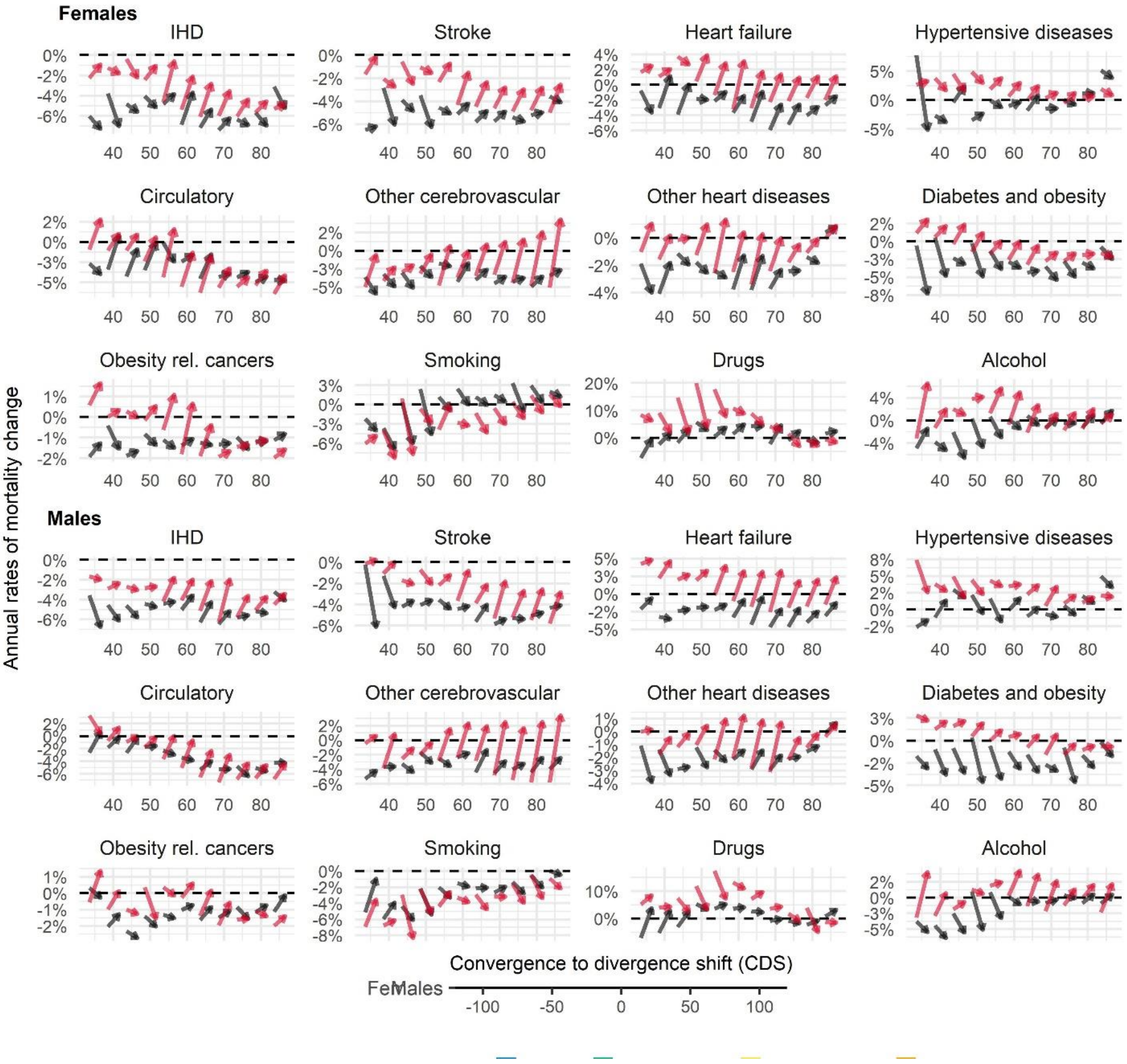

Figure S10. Differences in the average trends of age-specific annual change by cause during the periods of CVD gap convergence and divergence, excluding years with missing data. The same description as in the Figure S6 caption applies.

Robustness check 2: Estimates excluding countries with incomplete data

Figures S11-S14 reproduce the gap trend analyses but excluding the countries with incomplete data, i.e., Australia, Austria, Italy, and the UK.

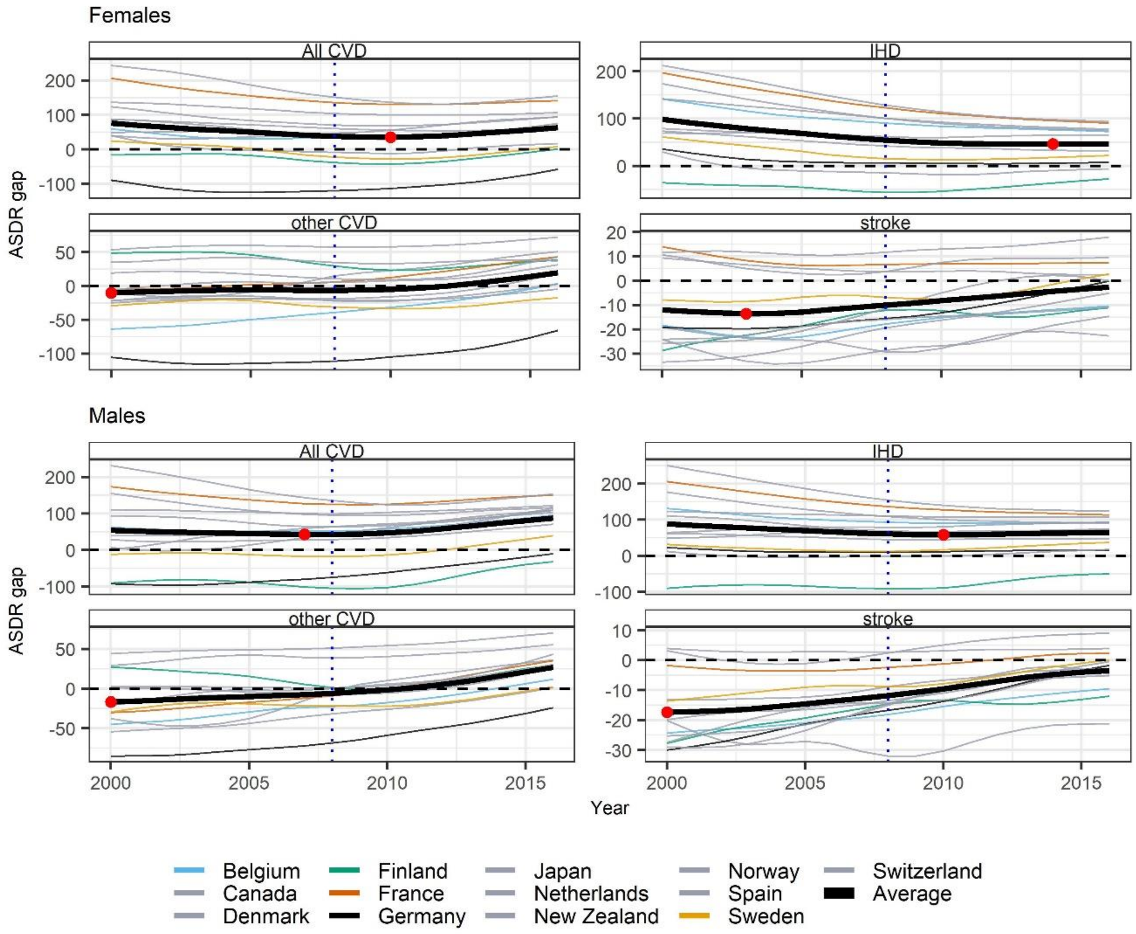

Figure S11. US-HLC gap in age-standardized death rates from CVD causes by sex, excluding HLC with incomplete data. The same description as in the Figure S1 caption applies.

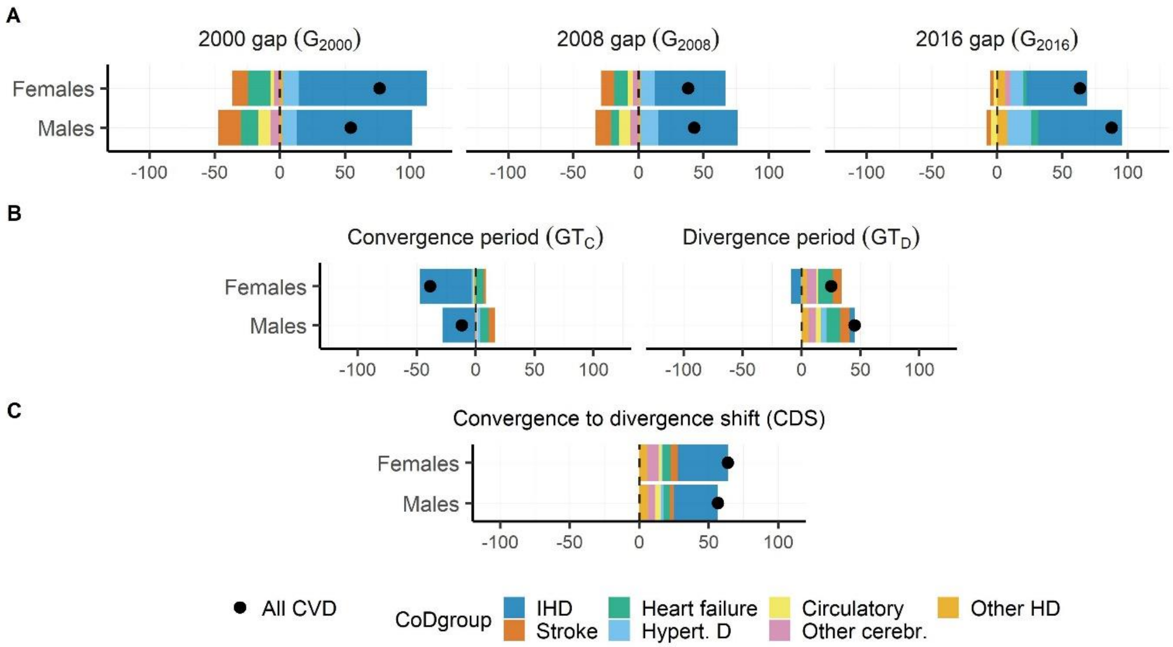

Figure S12. Cause-specific decomposition of overall age-standardized CVD death rates differences between the US and other HLC, excluding countries with incomplete data. The same description as in the Figure S3 caption applies.

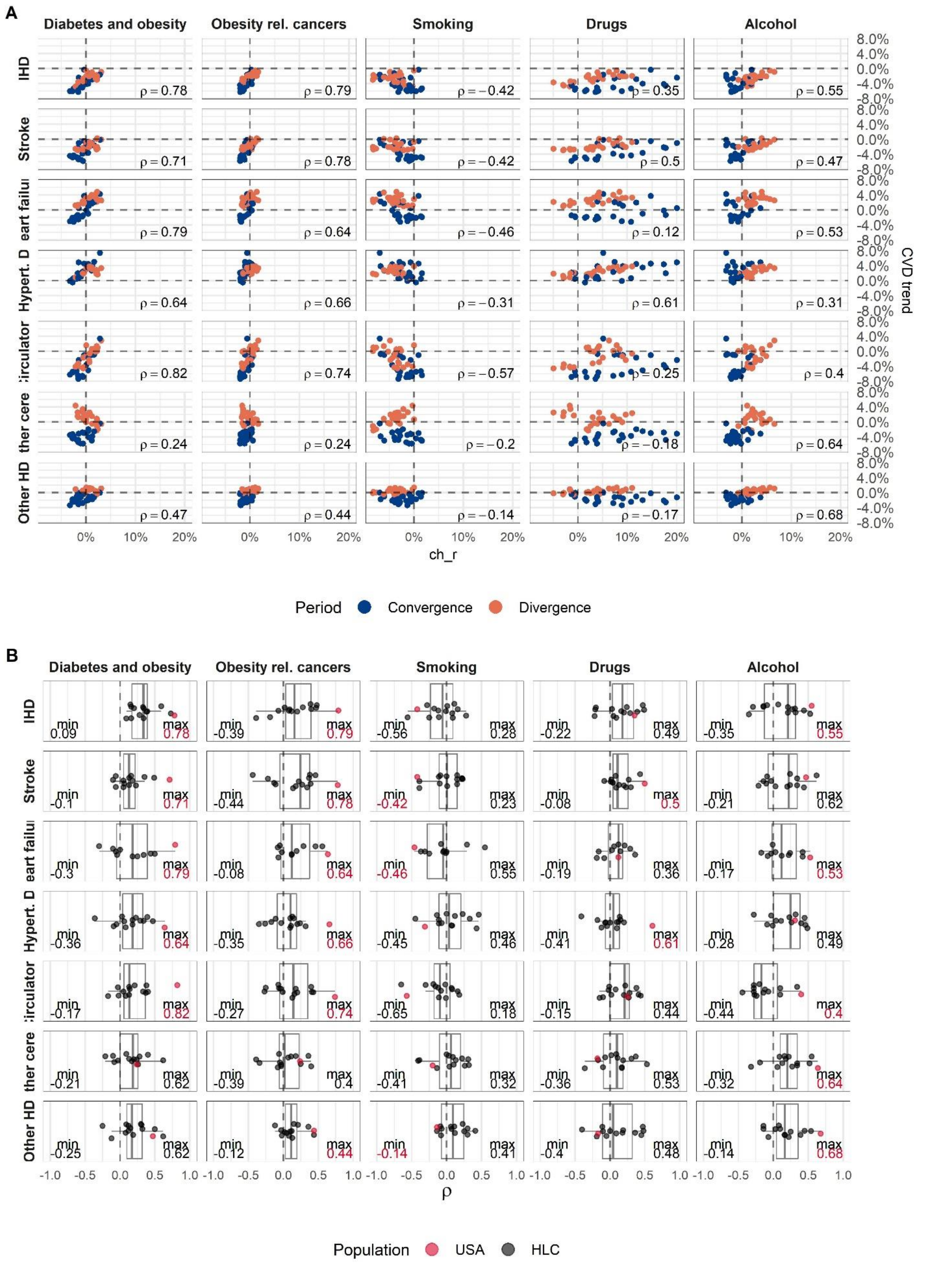

A
Diabetes and obesity
Obesity rel. cancers
Smoking
Drugs
Alcohol
IHD
Stroke
eart failu
Hypert. D
irculator
ther cere
Other HD
ρ = 0.78
ρ = 0.79
ρ = −0.42
ρ = 0.35
ρ = 0.55
ρ = 0.71
ρ = 0.78
ρ = −0.42
ρ = 0.5
ρ = 0.47
ρ = 0.79
ρ = 0.64
ρ = −0.46
ρ = 0.12
ρ = 0.53
ρ = 0.64
ρ = 0.66
ρ = −0.31
ρ = 0.61
ρ = 0.31
ρ = 0.82
ρ = 0.74
ρ = −0.57
ρ = 0.25
ρ = 0.4
ρ = 0.24
ρ = 0.24
ρ = −0.2
ρ = −0.18
ρ = 0.64
ρ = 0.47
ρ = 0.44
ρ = −0.14
ρ = −0.17
ρ = 0.68
8.0%
4.0%
0.0%
-4.0%
-8.0%
CVD trend
0%
10%
20%
ch_r
Period
Convergence
Divergence
B
Diabetes and obesity
Obesity rel. cancers
Smoking
Drugs
Alcohol
IHD
min 0.09 max 0.78
min -0.39 max 0.79
min -0.56 max 0.28
min -0.22 max 0.49
min -0.35 max 0.55
Stroke
min -0.1 max 0.71
min -0.44 max 0.78
min -0.42 max 0.23
min -0.08 max 0.5
min -0.21 max 0.62
eart failu
min -0.3 max 0.79
min -0.08 max 0.64
min -0.46 max 0.55
min -0.19 max 0.36
min -0.17 max 0.53
Hypert. D
min -0.36 max 0.64
min -0.35 max 0.66
min -0.45 max 0.46
min -0.41 max 0.61
min -0.28 max 0.49
irculator
min -0.17 max 0.82
min -0.27 max 0.74
min -0.65 max 0.18
min -0.15 max 0.44
min -0.44 max 0.4
ther cere
min -0.21 max 0.62
min -0.39 max 0.4
min -0.41 max 0.32
min -0.36 max 0.53
min -0.32 max 0.64
Other HD
min -0.25 max 0.62
min -0.12 max 0.44
min -0.14 max 0.41
min -0.4 max 0.48
min -0.14 max 0.68
-1.0
-0.5
0.0
0.5
1.0
ρ
Population
USA
HLC

Figure S13. Correlation of age- and sex-specific mortality trends between CVD and risk-related causes during the convergence and divergence periods, excluding countries with incomplete data. The same description as in the Figure S5 caption applies.

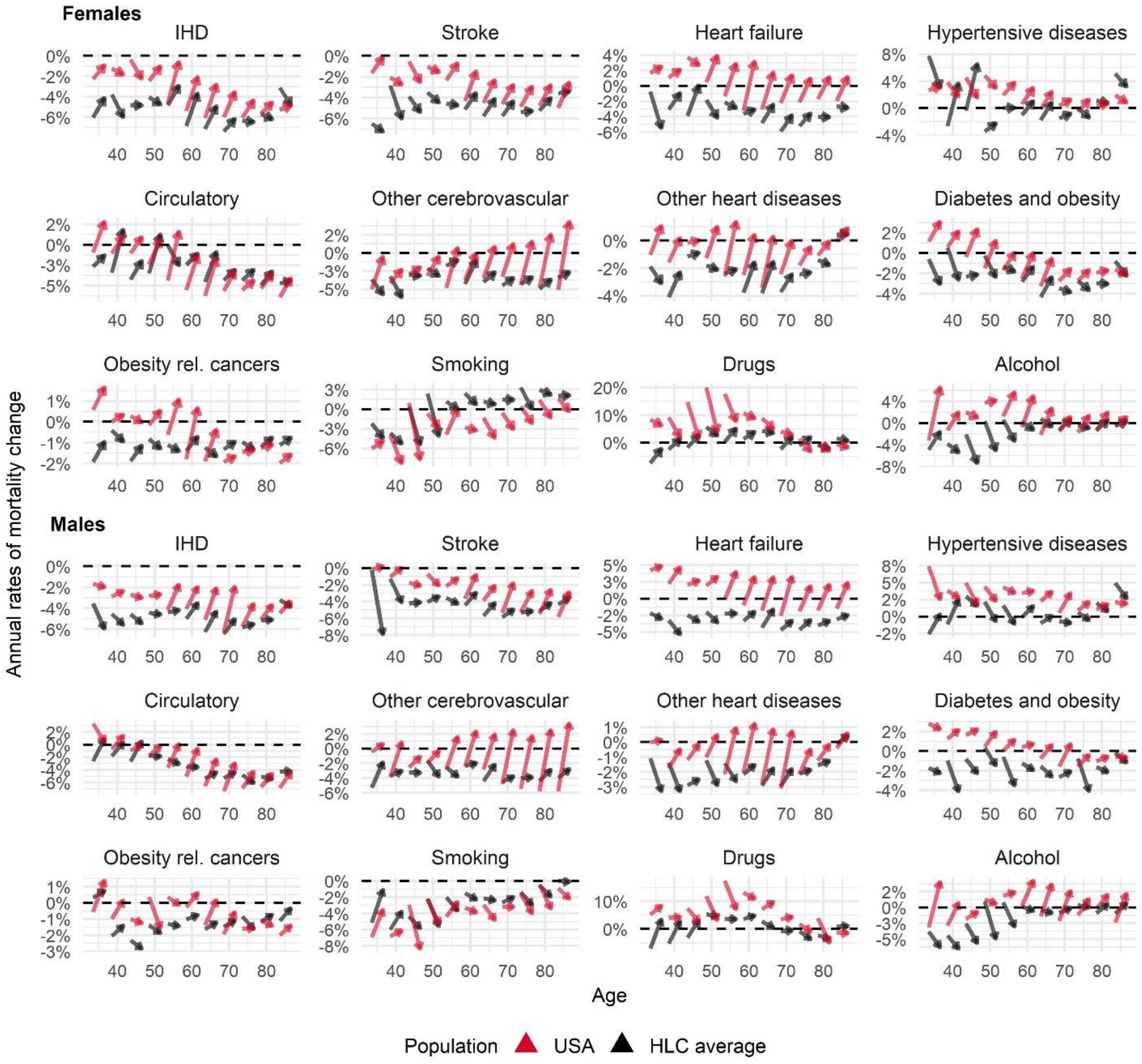

Figure S14. Differences in the average trends of age-specific annual change by cause during the periods of CVD gap convergence and divergence, excluding countries with incomplete data. The same description as in the Figure S6 caption applies.
